# A scoping review and research agenda: Psychological flexibility and wellbeing in organisations

**DOI:** 10.1111/aphw.70139

**Published:** 2026-03-12

**Authors:** Maree Roche, Josh W. Faulkner, Elise Callagher

**Affiliations:** ^1^ Business School University of Auckland Auckland New Zealand; ^2^ Clinical and Neuropsychology Department Te Herenga Waka–Victoria University of Wellington Wellington New Zealand

**Keywords:** Acceptance and Commitment Therapy, organisation, psychological flexibility, wellbeing

## Abstract

As workplaces increasingly recognise the importance of employee mental wellbeing, research into psychological flexibility and psychological inflexibility (PF/PI) has grown. PF, rooted in Acceptance and Commitment Therapy (ACT), is the capacity to pursue valued goals despite stressors and internal challenges, whereas PI reflects rigid attempts to avoid or control unwanted internal experiences, even when doing so undermines wellbeing or goal pursuit. Our systematic search across five databases identified 88 studies that had examined the relationship between PF/PI and wellbeing in workplace contexts. The findings indicate a surge in research since 2020, with PF (and, as such, PI) predominantly measured using the Acceptance and Action Questionnaire‐II (AAQ‐II). Most studies focus on mitigating negative wellbeing outcomes, such as burnout and psychological distress, rather than fostering positive wellbeing. PF/PI research is concentrated in high‐stress professions, particularly healthcare, with limited exploration in other sectors. Finally, PF/PI research is dominant in Western research. Recommendations for future research directions were established, including a clearer construct definition, development in measurement approaches, longitudinal and intervention‐based designs and broader occupational and cultural representation. Ultimately, this review highlights the need for a more nuanced and precise understanding of PF/PI to optimise its role in enhancing workplace wellbeing.

## INTRODUCTION

The upsurge in workplace complexities and disruptions continues to take a toll on the mental wellbeing of workers. As such, numerous calls are pressing for workplaces and researchers to ensure they prioritise mental wellbeing at work (Heron et al., 2024; Saraceno & Caldas De Almeida, 2022; WHO & ILO, 2022). Indeed, the OECD (2023) urges that wellbeing be a priority for workplaces, whereas other authors refer to the ongoing psychologically draining work conditions (Kelloway et al., 2023). Amid the ‘Great Exhaustion’ era of work (Verma et al., 2025, p. 2)—marked by escalating burnout rates, with one in five employees at risk—a different research lens advocates for the enhancement of positive wellbeing, suggesting that the focus on traditional deficit‐based approaches to mental wellbeing at work has proved inadequate (Randolph, 2021; Verma et al., 2025; WHO, 2022). The positive lens advocates that enhancing wellbeing and thriving is central to wellbeing research at work (Bauer & Jenny, 2012). Thus, both deficit and positive approaches to wellbeing appear central in enhancing and/or buffering the mental wellbeing of today's workforce.

Drawing from clinical psychology, one such means of buffering the toll of the Great Exhaustion era and increasing positive aspects of wellbeing is psychological flexibility (PF). Being able to pursue important goals, despite challenges, obstacles and demands, is fundamental to enhancing one's PF and resultant wellbeing (Hayes et al., 2006). Originally drawn from the third wave of behavioural interventions, Acceptance and Commitment Therapy (ACT) was designed to assist individuals in developing greater PF when encountering distressing experiences, while also addressing the tendencies associated with psychological inflexibility (PI) that can constrain effective coping. The ultimate aim of this intervention was to progress personal values while navigating the complexities and stress of life. Being able to do so not only aided PF but also mental health, and as such, PF is viewed as fundamental to wellbeing (Gloster et al., 2017; Kashdan & Rottenberg, 2010). ACT aims to achieve this by developing six interconnected skills: fostering awareness of ongoing experience, adopting a more open stance towards internal events, creating distance from unhelpful cognitive patterns, viewing the self from a more stable and contextual perspective and strengthening value‐guided behaviour (Hayes et al., 2006). Together, these skills comprise the Hexaflex framework of ACT, representing an intervention approach to address PF and resultant wellbeing.

In recent years, the interest in PF has increased immensely in the clinical domain (Doorley et al., 2020). It is therefore not surprising that this construct has also attracted the attention of organisational researchers and practitioners. PF has been shown to have positive outcomes on wellbeing (from both deficit and positive perspectives) for employees (Flaxman et al., 2023). For example, PF has been found to be associated with various indices of employees' mental health and work‐related functioning (e.g. Bond et al., 2013; Bond & Bunce, 2003; Kopperud et al., 2021; Vilardaga et al., 2011). This includes depression (Biglan et al., 2013), work stress (Blekić et al., 2023), PTSD (Bryan et al., 2015), substance misuse (Baker et al., 2022), burnout (Kent et al., 2019), resilience (Hendriks et al., 2021), job performance (Ding & Wang, 2022) and job satisfaction (Novaes et al., 2018). Clearly, PF may play an important role in employee wellbeing. The existing body of organisational research suggests many benefits of PF for the individual.

Given this, there has also been growing attention paid to using interventions that target PF within an organisational setting. Specifically, ACT‐based training and interventions have been adapted for employees, coaches and team leaders, as well as for use at the team and organisation levels (e.g. Bond et al., 2015; Prudenzi et al., 2021; Reeve et al., 2018). ACT interventions have also been applied to various organisational groups, including healthcare workers (Prudenzi et al., 2021), first responders (Baker et al., 2022), call centre employees (Bond & Flaxman, 2006), teachers (Galhardo et al., 2021) and government department employees (Lloyd et al., 2013). These interventions are typically programmes offering ACT‐based training over a small number of sessions to groups of employees (Prudenzi et al., 2021; Rudaz et al., 2017) across different formats, including online platforms, smartphone apps and bibliotherapy (e.g. Hofer et al., 2018; Ly et al., 2014). Taken together, ACT (and associated interventions) holds considerable promise as a psychological intervention within an organisational setting through the process of targeting PF/PI. PF is commonly discussed within the ACT framework, yet it is also informed by several other theoretical traditions. Bond and Flaxman (2006), for instance, advanced a *goal‐focused context sensitivity* account, describing PF as a regulatory mechanism that helps individuals allocate attention efficiently to behaviours that support their objectives. In organisational settings, PF has been conceptualised as a personal resource within the Job Demands–Resources model, where it functions to reduce strain and promote engagement. Additional work has highlighted links with resilience and recovery processes at work, suggesting that the capacity to remain open, responsive and guided by personal values supports effective unwinding from stress and facilitates adaptive re‐engagement (Kashdan & Rottenberg, 2010). PF also aligns with a range of organisational theories that seek to account for how features of the job and personal resources shape employees' wellbeing and effectiveness at work (for a review, see Flaxman et al., 2023). Given the findings of PF/PI and its alignment with wellbeing, there is a rationale and opportunity for ongoing research into the role of PF/PI not only in reducing the harmful impact of mental health but also in enhancing workplace wellbeing.

However, PF itself is not a straightforward concept regarding its definition, measurement, intervention or application in workplace environments. Early attempts to assess PF were largely borrowed from clinical intervention research, where measures were designed to evaluate change during ACT‐based therapy rather than to characterise naturally occurring flexibility in everyday work settings (McLoughlin & Roche, 2023). More recently, though, many self‐report measures of PF have been developed independently from empirically tested ACT (or related) interventions to assess and quantify PF (see Flaxman et al., 2023, for ACT intervention review). The measurement of PF has also evolved from unidimensional to multidimensional approaches. Although early measures treated PF as a unidimensional construct, only a few recent measures—such as the MPFI, CompACT and PsyFlex—capture PF as a multidimensional construct, as originally conceived in ACT (Cherry et al., 2021). Although the development of multidimensional measures within the clinical literature has been undertaken to overcome the limitations of the existing unidimensional measures (Cherry et al., 2021; Makriyianis et al., 2019; Rolffs et al., 2018), this work remains scattered for organisational researchers. Furthermore, the multidimensional measures of PF (e.g. MPFI, CompACT and PsyFlex) assess different dimensions of PF—that is, they do not all measure a uniform construct of PF (for a review, see Cherry et al., 2021, and Supporting Information: Table S1).

As an extension of this concept's muddiness, PF has been used to measure both PF and PI (Cherry et al., 2021; Makriyianis et al., 2019; Rolffs et al., 2018). PI has generally been defined as the ‘rigid dominance of psychological reactions over chosen values and contingencies in guiding action’ (Bond et al., 2011, p. 678). PI has been described as a broad process that contributes to vulnerability across many forms of psychological distress (Boulanger et al., 2010; Levin et al., 2014) and often also exhibits a related phenomenon, experiential avoidance (i.e. the tendency to *avoid c.f. accept*, unwanted thoughts and emotions), which is harmful and reduces wellbeing. Thus, *in*flexibility is a separate, albeit related construct to PF, but PF is based on cognitive behavioural interventions, most notably ACT (Hayes et al., 2011).

What, then, is PF? Originally conceptualised by Hayes et al. (2004), PF was defined as ‘the ability to change or persist with functional behavioural classes when doing so serves valued ends’ (p. 15). Although related to other constructs, PF is conceptually distinct. In contrast to adaptability, which concerns general adjustments to changing situations, PF involves being willing to acknowledge and make room for one's thoughts, emotions and sensations while continuing to move towards personally meaningful aims, even when those internal experiences are difficult (Kashdan & Rottenberg, 2010). Similarly, resilience refers to recovering from stress or adversity but does not necessarily involve the acceptance of ongoing internal discomfort during the pursuit of goals (Zautra et al., 2010). Coping styles also differ: Avoidance coping involves suppressing or escaping unwanted experiences, which is antithetical to PF (Kashdan et al., 2006), whereas proactive coping involves anticipating and planning for challenges, which may overlap with committed action but does not require experiential openness (Greenglass et al., 1999). PF has also been linked to personality dimensions such as lower neuroticism and higher openness to experience (Kashdan & Rottenberg, 2010; Rolffs et al., 2018), yet it is not reducible to traits, which reflect broad and stable tendencies. Instead, PF is a dynamic, context‐sensitive and modifiable process. Despite its importance, conceptual clarity has been hampered by a proliferation of measures that vary in constructs, dimensions and items, with some targeting PI rather than flexibility (Doorley et al., 2020; Kashdan et al., 2020). In response, Cherry et al. (2021) reviewed the literature and proposed three core components of PF: ‘(1) managing interference or distress, (2) taking action to regulate interference or distress and (3) behaving in ways that fit situational demands and support valued goals’ (p. 10). Nonetheless, definitional and measurement inconsistencies remain, highlighting the need for further theoretical and empirical refinement.

For organisational researchers, decisions regarding interventions and the measurement of PF are crucial. A key distinction in how the construct is developed and studied in the workplace lies in whether it is assessed as a state, trait or intervention outcome (i.e. Roche et al., 2021). As such, the measurement issues and complicating factors outlined above suggest that although there is potential for PF to enhance wellbeing, as found in clinical (and interventional) research, it is important that organisational researchers are clear on the meaning, measurement and use of PF and PI.

Moreover, regarding its components and definitions, wellbeing remains conceptually broad (Dodge et al., 2012; Goodman & Kashdan, 2025; Keyes & L., 2002; Östlund, 2024), reflected in organisational psychology as being ‘multifaceted that scaffolds many areas’ and transcending the individual, work and workplaces (Jarden et al., 2023, p. 2). From a generic, context‐free perspective, the hierarchical model of wellbeing builds upon previous conceptualisations of wellbeing existing within neuropsychological domains and as individual psychological attributes (Sirgy, 2019) by proposing a three‐tiered hierarchical framework (Goodman & Kashdan, 2025). The first tier of this framework encompasses the concept of hedonic wellbeing, understood as the happiness component of wellbeing describing the experience of pleasure via high positive and low negative affect and high satisfaction with life. Within the workplace—a context‐specific environment—this is also referred to as ‘subjective wellbeing’ (Diener et al., 2018; Jarden et al., 2023). The hierarchical model also includes eudaimonic wellbeing—a broad concept characterised by living life meaningfully (Goodman & Kashdan, 2025; Ryff, 1989) that includes dimensions such as engagement, meaning, growth, intrinsic motivation and calling (see Ryan & Deci, 2001). Within the workplace, this encompasses experiences such as autonomy, growth and self‐determination (Ryan & Deci, 2001). Finally, the hierarchical model incorporates social wellbeing via concepts such as belonging, relationship strength and social connection—notions that, in the workplace context, encompass team‐, group‐ and leadership‐level relationships (see Goodman & Kashdan, 2025; Jarden et al., 2023).

In contrast, there exist various other work‐specific wellbeing definitions that encompass much broader concepts than those outlined by the three‐tiered framework. These definitions include organisational practices, systems and overarching, external social contexts, such as legislation and politics (see Nielsen et al., 2018). Nevertheless, across the various conceptualisations, the central ideas of wellbeing at work remain similar, encompassing working conditions, work factors, organisational design and work stressors (Jarden et al., 2023; Laine & Rinne, 2015; Nielsen et al., 2018). Definitions of wellbeing that approach the concept from a broader perspective enable the inclusion of home–work and work–home complexities—stressful (and positive) events that may transfer from work to home and vice versa (Sonnentag et al., 2021; ten Brummelhuis et al., 2014).

A further challenge of defining workplace wellbeing concerns the relationship between positive and negative wellbeing, which evidence suggests is complex. That is, although the ‘end points’ (such as mental ill health or flourishing) can be differentiated, research lacks a consensus with respect to the exact definitions and confines of wellbeing versus ill‐being, necessitating clarification of this conceptual continuum to eliminate ambiguity (Kelloway et al., 2023; Keyes & L., 2002; Sonnentag et al., 2023). In a practical sense, individuals may simultaneously experience a state of positive mental health alongside mental health problems. This may manifest as general satisfaction and happiness in life in tandem with distress and difficulty at work or, conversely, meaningful engagement at work alongside generalised anxiety in life. This dichotomy highlights the importance of considering wellbeing as a continuum of states that can simultaneously co‐exist.

The concept of wellbeing is broad and sparks significant disagreement regarding its definition (Östlund, 2024); scholars' interpretations range from context‐free, hierarchical conceptualizations to context‐specific definitions that encompass the work itself, the workplace and the environment. Despite its derivation from a positive psychology perspective, wellbeing is thought to exist on a continuum, with various constructs coexisting simultaneously. This broad, all‐encompassing formulation has implications when researchers endeavour to understand research on PF/PI and workplace wellbeing. Therefore, this review adopted a broad, encompassing definition of wellbeing.

In summary, given the extent and breadth of the literature regarding the potential role of PF/PI in mental and work wellbeing and its process of change via ACT, along with the diversity of measures and variables, we suggest the time is right to provide a coherent overview of PF/PI within organisations in order to guide future wellbeing research. This is the objective of the current study. The aim of this paper is to examine emerging issues regarding PF/PI and wellbeing at work and thus provide insight into this developing field for readers and researchers. Thus, we conducted a scoping review of studies that examined the relationship between PF/PI and wellbeing in organisational settings. To date, only one prior scoping review has synthesised psychological flexibility and related constructs more broadly (Cherry et al., 2021), examining diverse conceptualisations and measures of psychological flexibility across clinical and non‐clinical literature, highlighting definitional overlaps and inconsistencies. However, their synthesis was not specific to organisational contexts and did not focus on workplace wellbeing outcomes. Given the diversity of definitions, measures and outcomes associated with PF/PI in the literature, a scoping review methodology was deemed the most appropriate. Unlike systematic reviews, which address narrowly defined questions and assess quality and effect sizes, scoping reviews are designed to map heterogeneous literature, clarify conceptual boundaries and identify research gaps (Arksey & O'Malley, 2005; Levac et al., 2010). This approach aligns with our objective to provide a broad synthesis of how PF/PI has been studied in organisational contexts and highlight areas for future investigation.

Specifically, this review maps the existing research on PF/PI and identifies key concepts, measures, trends and issues in measurement, industries, occupations, countries and wellbeing outcomes. We identify gaps and complexities, ultimately aiming to guide future research. Our scoping research questions are as follows:

1. How is PF/PI currently being measured and defined in organisational settings?
2. What specific types of organisational settings are PF/PI currently being examined in?
3. What workplace wellbeing outcomes are being examined in terms of their relationship with PF/PI in organisational settings?

## METHOD

### Scoping review

A scoping review is used to map the key concepts underpinning a research area (Shaw et al., 2010). It is often used when the topic is heterogeneous and the aim is to map a body of literature, identify key concepts and highlight gaps (Pham et al., 2014). This review was informed by Arksey and O'Malley's five‐stage scoping review methodology (Arksey & O'Malley, 2005) and also used strategies recommended by Levac et al. (2010). The PRISMA extension guidelines for scoping reviews (PRISMA‐ScR; Tricco et al., 2018) were used. The ethics statement is as follows: Following institutional guidelines, as the data were already in the public domain and were being used for systematic review only, the project did not require institutional ethical approval.

### Search strategy

The search strategy was comprised of three concepts: construct, outcome and population/context. The construct was PF and PI, which also included the following terms: cognitive fusion, experimental avoidance and ACT. The outcome was wellbeing and included the following terms: burnout, stress, work stress, stress management, psychological stress, resilience, emotion regulation, job satisfaction, coping, occupational stress, quality of life, compassion fatigue, work engagement, wellbeing, mental health and depression. The population/context was organisational settings and included the following terms: organisational, organisational, employee, management, leadership, leader, workplace, work, staff and personnel. See Supporting Information (Table S2) for a summary of the search terms.

### Information sources

The following databases were searched: MEDLINE, PsycInfo, Scopus, Embase, Taylor & Francis and Web of Science. These databases were selected to ensure broad and complementary coverage of the most relevant literature regarding the review question. MEDLINE and Embase were included to capture research indexed within health and medical sciences. PsycInfo was selected for its comprehensive coverage of psychology and behavioural sciences, including organisational psychology. Scopus, Web of Science and Taylor & Francis were included, given their multidisciplinary indexing of high‐quality journals across psychology, health and organisational sciences. This combination was therefore considered to provide wide coverage across clinical, psychological and organisational domains. In addition, citation searches were undertaken to identify further relevant studies. There were no time restrictions specified for article inclusion. The search took place from December to January 2023 and was updated on 05 November 2024.

### Eligibility criteria

The following inclusion criteria were applied:

1. Population: The sample used in the study was identified as an adult (18 years and older) employee group in any profession working in an organisational setting.
2. Measured psychological flexibility/inflexibility: The study included and reported at least one measure of PF.
3. Outcome measures: The study reported a relationship between PF/PI and at least one outcome measure of wellbeing.
4. Peer reviewed: The study was published in a refereed journal. This was to ensure a high quality of articles deemed appropriate for publication by the scientific community.

The following exclusion criteria were used:

1. Study design: Single case studies were excluded as these studies were deemed insufficient to enable a holistic understanding of PF/PI within the organisational setting in which it was being examined.
2. Study focus: Studies that examined flexibility outside the scope of PF/PI were also excluded, that is, work flexibility, flexible work hours or flexible workspace.
3. Reporting change in variables alone: Intervention studies that only reported a change in PF/PI and/or workplace wellbeing outcomes associated with an intervention were also excluded.

### Study selection and data collection process

The identified papers were imported into Covidence, and duplicates were removed. Screening consisted of two stages: title and abstract screening and full‐text review. The initial screening was completed by the principal reviewer (PR) and two secondary independent reviewers (SIRs). Screening included reviewing article titles and abstracts against eligibility criteria. An initial learning phase occurred where the PR screened a small batch of papers (*n* = 10) and then discussed any discrepancies in the screening decisions with the SIRs. This process was repeated until consistency was reached in the PR's decision‐making with the other researchers. During the initial screening, any unclear decisions were flagged and then discussed with both SIRs. We erred on the side of inclusion if we could not reach a unanimous exclusion decision. For full‐text screening, the same process was repeated; however, the inclusion of a study required consensus from all of the authors.

### Data extraction and synthesis

Following title and abstract screening, full‐text articles were reviewed. Data extraction from full‐text articles included the author, title, study design, study location, date, sample size and occupational group. The tool used to measure PF/PI and how it was defined in the study was also extracted, as was the specific workplace wellbeing construct and the tool used to measure it. The findings pertaining to the strength of the correlation between the measure of PF/PI and the specific workplace wellbeing measure were extracted, along with other relevant non‐correlation findings (e.g. mediation or moderation effects). A bespoke data extraction tool was developed iteratively and piloted on a small number of studies. The second researcher carried out data extraction for a randomly allocated selection of papers. The data extraction of the two researchers was compared, and any inconsistencies were discussed in order to reach a consensus. Data were extracted and uploaded into a data dictionary by the PR. Separate data dictionaries were used for each workplace wellbeing construct. Frequency counts were used to summarise study characteristics (i.e. study location and year) and the organisation setting examined in each study. We also counted the number of individual measures of PF/PI. For this analysis, percentage frequencies of the measures of PF/PI were computed. We then grouped the measures of PF/PI based on context, that is, generic or context‐specific measures. Generic measures of PF assess the individual's general capacity to accept internal experiences and pursue valued action across life domains. By contrast, context‐specific measures adapt this framework to a particular setting, embedding items within domain‐relevant stressors (e.g. work dissatisfaction, classroom stress or weight‐related stigma).

A similar approach was undertaken for the wellbeing outcomes within the context of PF/PI in organisational settings. The wellbeing outcomes were categorised into variables based on their valence (positive or negative) and context (general or occupational wellbeing).

## RESULTS

### Study selection

**FIGURE 1:**
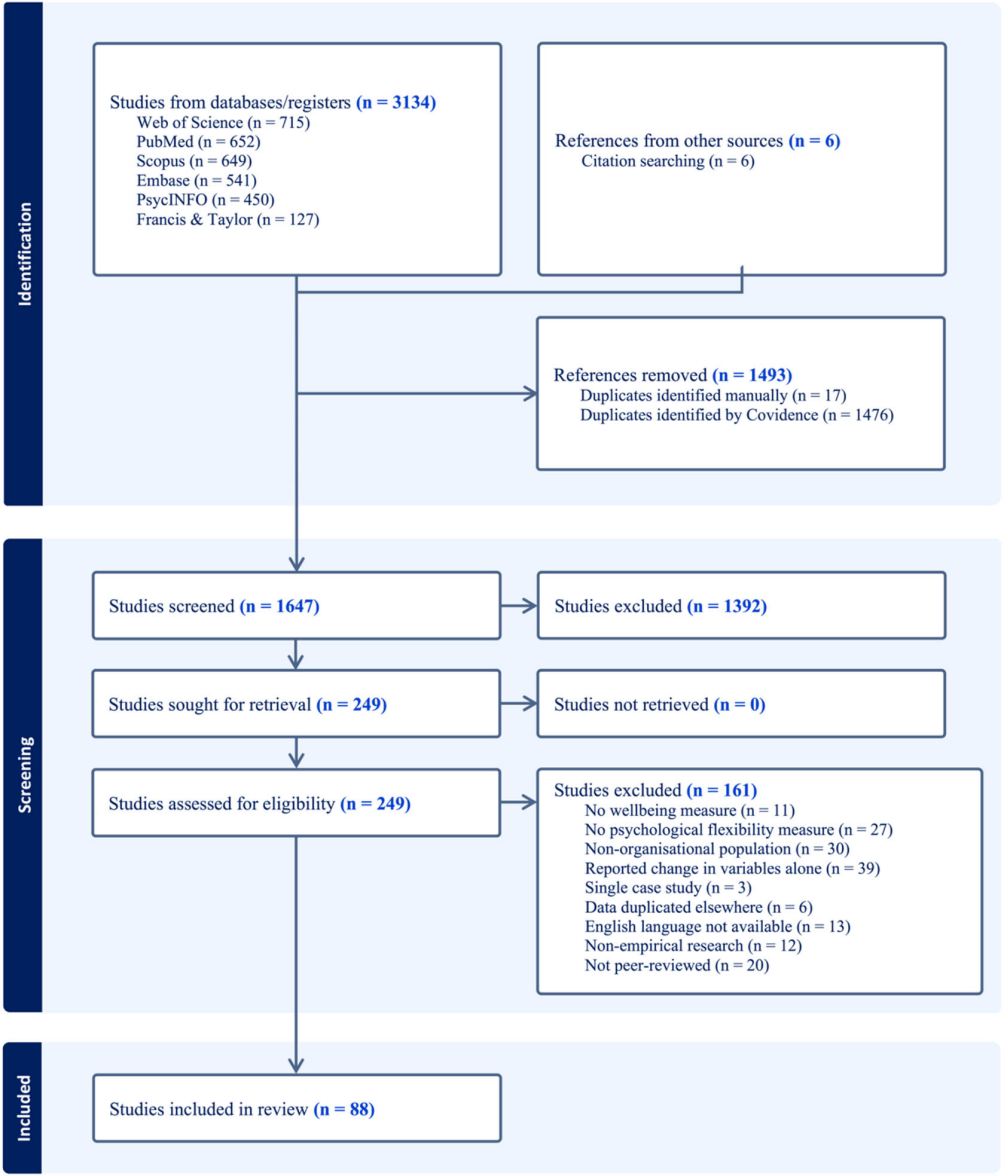
PRISMA‐ScR diagram.

The search yielded 3134 articles (MEDLINE [652], PsycInfo [450], Scopus [649], Embase [541] and Web of Science [715]; see Figure 1). Additionally, six articles identified through the citation search were included in the screening process. Removing duplicates resulted in 1647 articles. Titles and abstracts were screened against eligibility criteria, resulting in 249 full‐text articles to review. Of these articles, 161 were excluded for the following reasons: no wellbeing measure (*n* = 11), no PF measure (*n* = 27), non‐organisational population (*n* = 30), reported change in variables alone (*n* = 39), single case study (*n* = 3), data duplicated elsewhere (*n* = 6), English language not available (*n* = 13), not empirical research (*n* = 12) and not peer‐reviewed (*n* = 20). Thus, a total of 88 articles were included in the review.

### Study characteristics

**FIGURE 2:**
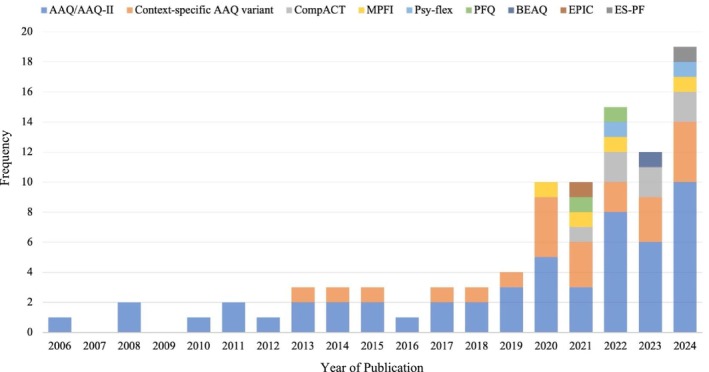
Use of psychological flexibility/psychological inflexibility measures by year of publication. *Note*: Context‐specific AAQ variants include the WAAQ, TAAQ and AAQW‐R.

Of the 88 articles included, the sample size ranged from 21 to 4867 (*M* = 367, SD = 569). The majority of studies were of a cross‐sectional design (*n* = 68, 77.3%), with a minority being intervention studies (*n* = 14, 15.9%), daily diary studies (*n* = 2, 2.3%) or studies adopting a longitudinal design (*n* = 3, 3.5%). A single study adopted both a cross‐sectional design and a longitudinal design for a subset of their sample (*n* = 1, 1.1%). As illustrated in Figure 2, the first study exploring the relationship between PF/PI and wellbeing in an organisational context was published in 2006. A significant increase in studies occurred in 2020, coinciding with the global COVID‐19 pandemic. Notably, 25 studies (28.4%) examined the link between PF/PI and wellbeing within the COVID‐19 context. This upward trend has continued, with the highest number of studies being published in 2024 (see Figure 2).

### Measures of PF/PI

Twelve distinct measures were used to assess PF/PI across the articles reviewed (see Table 1). Most studies used the Acceptance and Action Questionnaire (the AAQ or AAQ‐II; *n* = 54, 60.88%) or an AAQ variant (i.e. the WAAQ, TAAQ or AAQW‐R; *n* = 22, 23.40%). Six studies employed two measures of PF/PI (Bond et al., 2013: WAAQ and AAQ‐II; Bravo et al., 2023: WAAQ and AAQ‐II; Hinds et al., 2015: AAQ‐II and TAAQ; Koh et al., 2024: TAAQ and AAQ‐II; Tynan et al., 2022: CompACT and AAQW‐R; Xu et al., 2018: WAAQ and AAQ‐II).

**TABLE 1:** Measures used to assess PF/PI across the articles reviewed and their frequency.

Measure	Description	Generic or context	Frequency count	Frequency %
Acceptance and Action Questionnaire (AAQ)	A 9‐item self‐report measure of experiential avoidance and psychological flexibility, focusing on how individuals respond to their internal experiences, such as thoughts and emotions	Generic	5	5.62%
AAQ‐II	A 7‐item refined version of the original AAQ with an ongoing focus on assessing experiential avoidance and psychological flexibility	Generic	49	55.06%
Work‐related Acceptance and Action Questionnaire (WAAQ)	A 7‐item variant of the AAQ‐II; however, it is designed to assess *psychological flexibility* in the workplace. The WAAQ evaluates the extent to which employees accept or are willing to experience work‐related thoughts and emotions (e.g. stress or dissatisfaction).	Context	18	19.15%
Teacher Acceptance and Action Questionnaire (TAAQ)	A 9‐item variant of the AAQ‐II, designed to assess the context of teaching. Specifically, it measures how teachers respond to work‐related thoughts, emotions and challenges, such as stress or pressure, and their ability to remain engaged in meaningful teaching practices while staying aligned with their values.	Context	3	3.19%
Acceptance and Action Questionnaire for Weight‐Related Difficulties–Revised (AAQW‐R)	A 10‐item variant of the AAQ‐II, revised from the AAQW (Lillis & Hayes, 2008), in the context of difficulties related to eating, weight and physical activity. Specifically, it assesses the use of food to cope with negative emotions, weight as a barrier to valued living and weight‐related internalised stigma.	Context	1	1.06%
Comprehensive assessment of Acceptance and Commitment Therapy processes (CompACT)	A 24‐item self‐report measure of psychological flexibility designed to assess the six core subprocesses of psychological flexibility, namely, acceptance, cognitive defusion, present‐moment awareness, self‐as‐context, values and committed action	Generic	7	7.45%
Multidimensional Psychological Flexibility Inventory (MPFI)^a^	A 60‐item comprehensive self‐report of psychological flexibility (30 items) and inflexibility (30 items) across 12 corresponding subprocesses. It evaluates how individuals exhibit psychological flexibility in their thoughts, emotions and behaviours, as well as their ability to engage in value‐driven actions.	Generic	4	4.26%
Psy‐Flex	A 6‐item self‐report measure of psychological flexibility that assesses the six core subprocesses of psychological flexibility with high temporal specificity (i.e. in the last week)	Generic	2	2.13%
Psychological Flexibility Questionnaire (PFQ)	An 18‐item self‐report measure of psychological flexibility that assesses five dimensions, namely, q positive perception of change, a characterisation of the self as flexible, self‐characterisation as open and innovative, perception of reality as dynamic and changing and a perception of reality as multifaceted	Generic	2	2.13%
Experiential Psychological Inflexibility in Context (EPIC)	A 23‐item self‐report measure of psychological inflexibility. It focuses on how individuals react to their internal experiences across various contexts and how this impacts their ability to make value‐driven actions.	Generic	1	1.06%
Brief Experiential Avoidance Questionnaire (BEAQ)	A 6‐item self‐report measure designed to assess experiential avoidance. It focuses on how individuals react to distressing experiences and their inclination to engage in avoidance behaviours rather than accepting or confronting these experiences.	Generic	1	1.06%
The Euthymia Scale–Psychological Flexibility (ES‐PF)	A 5‐item self‐report measure assessing psychological flexibility. It is derived from the Euthymia Scale, which evaluates euthymia and psychological wellbeing. It evaluates stress resilience, frustration tolerance and adaptability to changing circumstances.	Generic	1	1.06%
Context				
Generic PF/PI Measure	Generic measures of PF/PI are used across various contexts and populations. Generic psychological flexibility measures assess an individual's ability to adapt to and manage internal experiences (such as thoughts, emotions and sensations) while engaging in actions that align with personal values, even in the presence of discomfort. In contrast, psychological inflexibility reflects rigid attempts to avoid, suppress or become entangled with such experiences in ways that undermine valued action. These measures typically evaluate key processes such as acceptance, cognitive defusion, mindfulness and committed action. Examples include the AAQ‐II, CompACT and MPFI.	72		76.60%
Context‐Specific PF/PI Measure	Context‐specific versions of PF/PI measures are adapted to assess these constructs within particular domains, such as work, teaching or specific life situations. Psychological flexibility refers to the ability to remain open to internal experiences while pursuing valued actions, whereas psychological inflexibility reflects rigid avoidance or entanglement with these experiences in ways that hinder valued behaviour. These measures retain the core PF processes (e.g. acceptance, cognitive defusion and committed action) but anchor them in situational demands. Examples include the WAAQ, TAAQ and AAQW‐R.	22		23.40%

*Note*: Six studies employed two measures of psychological flexibility (Bond et al., 2013: WAAQ and AAQ‐II; Bravo et al., 2023: WAAQ and AAQ‐II; Hinds et al., 2015: AAQ‐II and TAAQ; Tynan et al., 2022: CompACT and AAQW‐R; Xu et al., 2018: WAAQ and AAQ‐II; Koh et al., 2024: TAAQ and AAQ‐II).

^a^ Two studies (Wang et al., 2024; Young et al., 2022) only used the 30‐item psychological flexibility subscale of the MPFI.

The measures could be broadly categorised into two contexts: generic and context‐specific measures of PF/PI. Generic measures (*n* = 72, 76.60%) were designed to assess PF/PI across various situations and populations. These included instruments such as the AAQ‐II, CompACT and MPFI. Context‐specific measures (*n* = 22, 23.40%) were adapted to assess PF/PI within particular domains, such as the workplace. The WAAQ (*n* = 18, 19.15%) was the most commonly used context‐specific measure, followed by the TAAQ (*n* = 3, 3.19%). Notably, although the WAAQ was designed to assess work‐related PF (Bond et al., 2013), some studies describe it using inflexibility/avoidance terminology; we coded constructs based on how authors described the measure.

Figure 2 shows the use of PF/PI measures over time within the included studies. Most notably, the AAQ/AAQ‐II and its variants have continued to dominate the field. From 2006 to 2016, studies almost exclusively relied on the AAQ/AAQ‐II, with a small but growing use of context‐specific variants. Over time, there has been increased use of multidimensional measures of PF/PI, that is, MPFI or CompACT. Although there was a marked increase in research activity from 2019 onwards, this growth was not accompanied by a shift towards multidimensional measures of PF/PI, and the AAQ‐II continues to be the most widely used measure. The use of context‐specific measures and AAQ variants developed for particular domains (e.g. the workplace or teaching) has become more prevalent, suggesting growing recognition of the need to consider domain‐specific measurements of PF/PI.

**FIGURE 3:**
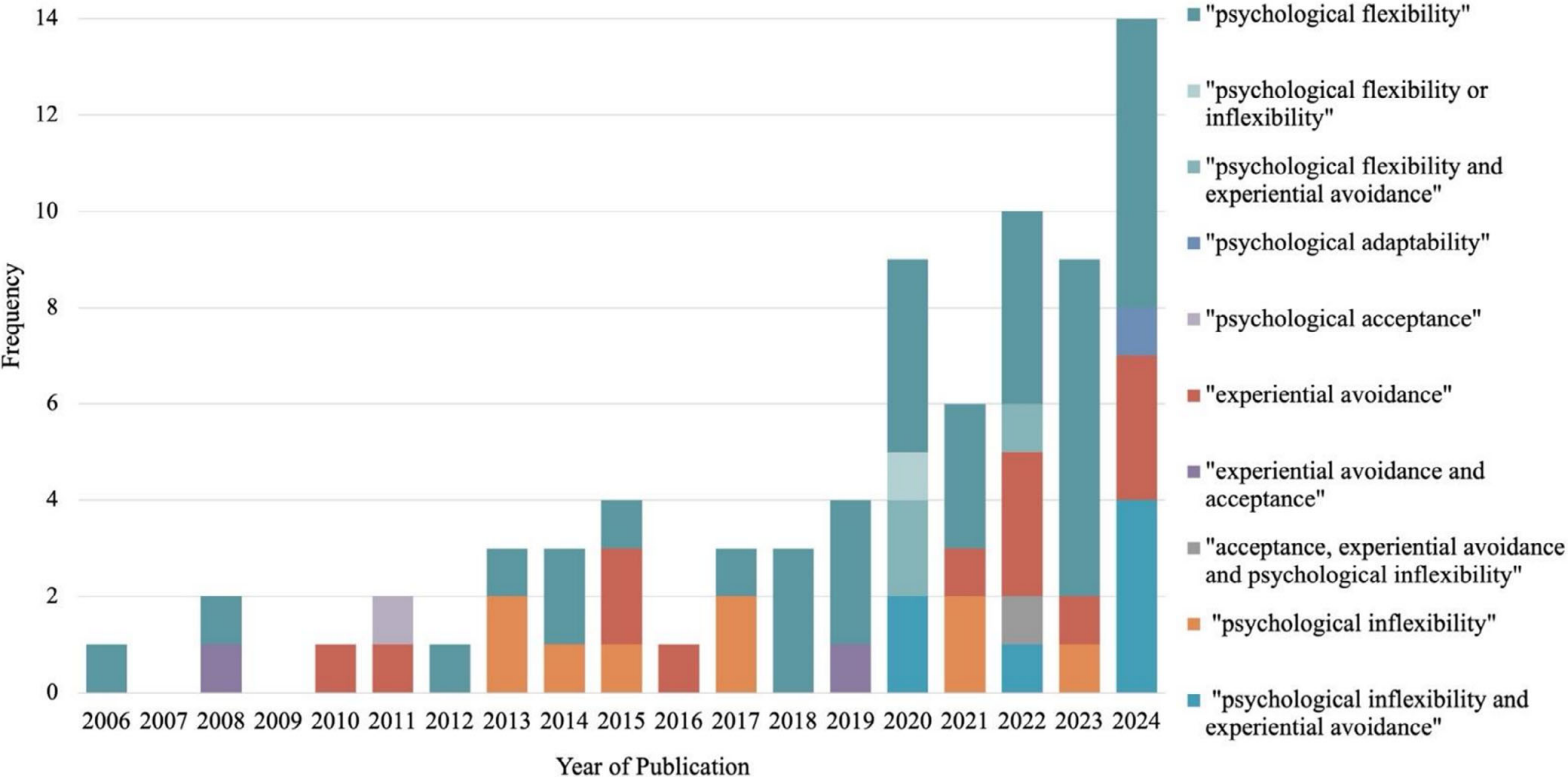
Constructs defined as being measured by the AAQ‐II by year of publication.

Given the predominant use of the AAQ‐II in the articles reviewed, we also sought to determine the constructs these studies identified longitudinally as being measured by the AAQ‐II. As shown in Figure 3, the AAQ‐II has been described as measuring a range of constructs. The most common of these was PF, followed by experiential avoidance. Some studies described the AAQ‐II as measuring multiple constructs, such as experiential avoidance and acceptance; acceptance, PI and experiential avoidance; or even PF and inflexibility. Some studies even described the AAQ‐II as a measure of psychological adaptability and psychological acceptance. Of note, we also mapped the constructs defined by the AAQ‐II over time. In 2024, there was greater use of the PI construct, as well as experiential avoidance. However, it is difficult to determine if this is a trend or an outlier, as these terms were used less frequently in 2023. What is still evident, however, is that in recent years, there was still a high proportion of studies that described the AAQ‐II as being a measure of PF.

### Outcome measures

**FIGURE 4:**
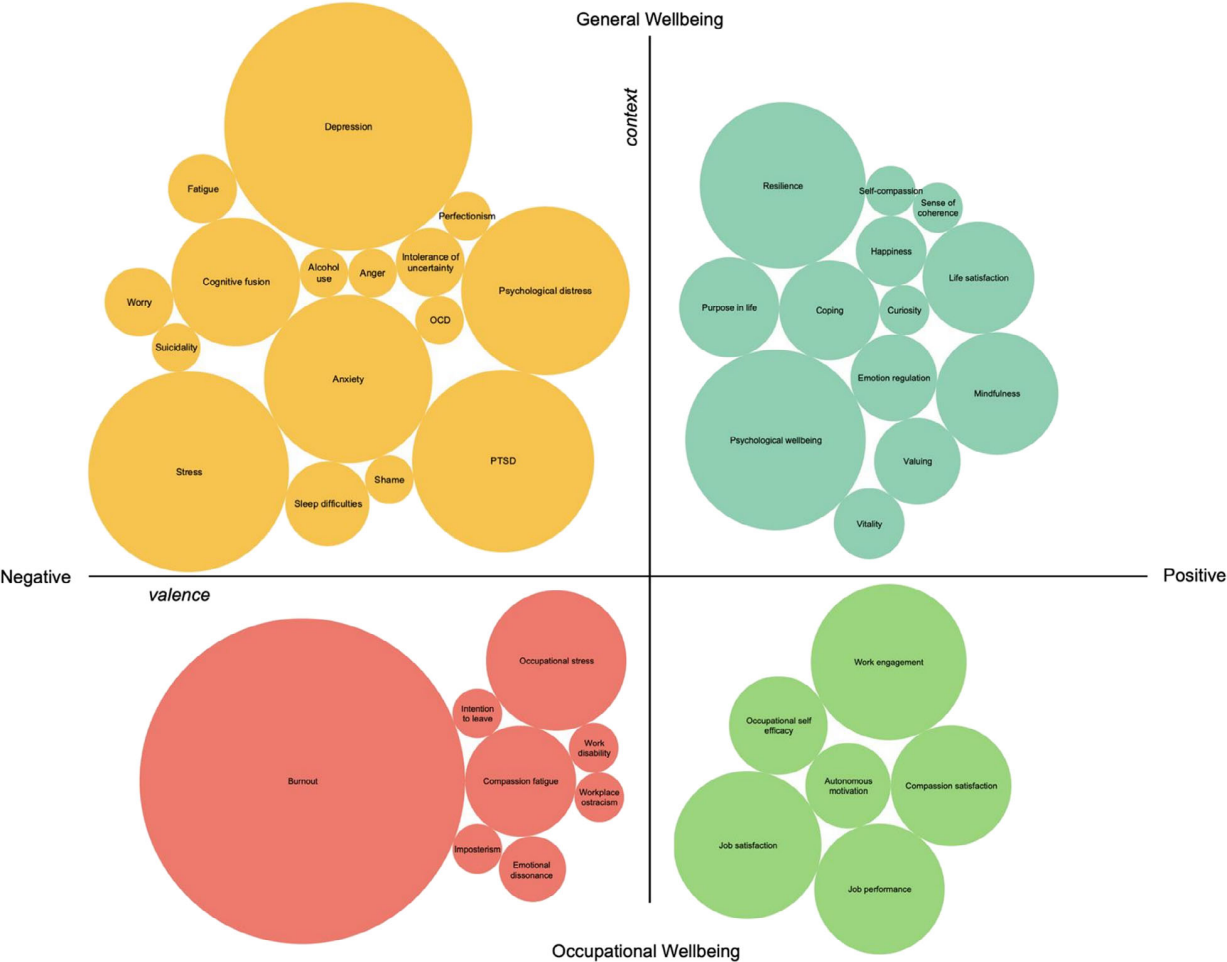
Wellbeing outcomes examined by visualisation matrix based on emotional valence and context. *Note*: Bubble size represents measurement frequency across the 88 studies (see Tables 2 and S3–S6). The axes represent categorical dimensions rather than continuous scales.

We also identified how wellbeing was examined in relation to PF/PI within an organisational setting. Using the broad understanding of wellbeing outlined above, the outcome measures were categorised into variables based on their valence (positive or negative) and context (general/generic or occupational wellbeing). These are summarised in Table 2 and Figure 4, with these variables visually organised into a bubble chart in which the sizes of the bubbles represent the frequency of studies measuring this outcome. A detailed summary of the measures used to assess these wellbeing variables is included in the Supporting Information (Tables S3–S6).

**TABLE 2:** Summary of the wellbeing outcomes examined and proportion of studies including each outcome (*N* = 88).

Wellbeing domain	Variable	Studies (*n*)	% of studies
Negative general wellbeing	Depression	26	29.5%
	Stress	17	19.3%
	Post‐traumatic stress disorder (PTSD)	14	15.9%
	Psychological distress	12	13.6%
	Anxiety	12	13.6%
	Cognitive fusion	7	8.0%
	Sleep difficulties	3	3.4%
	Fatigue	2	2.3%
	Intolerance of uncertainty	2	2.3%
	Worry	2	2.3%
	Perfectionism	1	1.1%
	Alcohol use	1	1.1%
	Anger	1	1.1%
	Suicidality	1	1.1%
	Shame	1	1.1%
	Obsessive compulsive disorder (OCD)	1	1.1%
Positive general wellbeing	Psychological wellbeing	13	14.8%
	Resilience	11	12.5%
	Mindfulness	10	11.4%
	Life satisfaction	5	5.7%
	Coping	4	4.5%
	Valuing	3	3.4%
	Emotion regulation	3	3.4%
	Happiness	2	2.3%
	Vitality	2	2.3%
	Sense of coherence	1	1.1%
	Self‐compassion	1	1.1%
	Curiosity	1	1.1%
Positive occupational wellbeing	Work engagement	10	11.4%
	Job satisfaction	9	10.2%
	Job performance	7	8.0%
	Compassion satisfaction	6	6.8%
	Occupational self‐efficacy	4	4.5%
	Autonomous motivation	3	3.4%
Negative occupational wellbeing	Burnout	43	48.9%
	Occupational stress	8	9.1%
	Compassion fatigue	5	5.7%
	Emotional dissonance	2	2.3%
	Workplace ostracism	1	1.1%
	Intention to leave	1	1.1%
	Work disability	1	1.1%
	Imposterism	1	1.1%

*Note*: Percentages reflect the proportion of included studies (*N* = 88) that assessed each wellbeing outcome. Studies could assess more than one outcome; therefore, percentages do not sum to 100%.

The analysis of outcome measures reveals several notable patterns regarding how wellbeing is conceptualised and measured in PF/PI research. Negative occupational wellbeing measures, particularly burnout (*n* = 43, 16.5%), dominated the literature, appearing in nearly half of all studies reviewed. This focus on burnout far exceeded the attention given to other negative occupational outcomes, such as occupational stress (*n* = 8, 3.1%) and compassion fatigue (*n* = 5, 1.9%). Positive outcome measures appeared less frequently in the literature. Within occupational contexts, work engagement (*n* = 10, 3.8%) and job satisfaction (*n* = 9, 3.5%) were the most common positive measures.

In terms of general wellbeing measures, depression (*n* = 26, 10%) was the most frequently assessed negative wellbeing outcome, followed by stress (*n* = 17, 6.5%) and PTSD (*n* = 14, 5.4%). The high prevalence of these measures suggests a strong focus on clinical or disorder‐based conceptualisations of wellbeing in organisational settings. Regarding general positive wellbeing, overall psychological wellbeing (*n* = 13, 5%) and resilience (*n* = 11, 4.2%) were most frequently assessed.

Some outcomes showed conceptual overlap but were measured as distinct constructs. For example, stress was measured both as a general construct and with occupation‐specific measures of stress, suggesting different conceptualisations of stress depending on the context. Similarly, compassion‐related measures appeared in both positive (compassion satisfaction, *n* = 6) and negative (compassion fatigue, *n* = 5) forms, particularly in healthcare settings.

### PF/PI as a mediator and moderator

Beyond only examining direct bivariate relationships, 33 studies (37.5%) also employed mediation or moderation frameworks to examine PF/PI. Twenty‐five studies positioned PF/PI as a mediating variable, largely testing whether it accounts for the relationships between workplace antecedents (e.g. job demands and social support) and wellbeing outcomes (e.g. burnout, depression and work engagement). Of these, 16 positioned PF as the mediator, whereas nine attributed PI to this role. These studies used various statistical approaches, including structural equation modelling, random intercept cross‐lagged panel models and bootstrapped mediation analyses. See Supporting Information for an overview of these studies (see Table S6).

Eleven studies positioned PF/PI as a moderating variable, whereby nine positioned PF as the moderator and only one positioned PI in this role. Two studies tested PF as both a mediator and a moderator.

### Organisational setting

We analysed the organisational sectors in which PF/PI was studied. As shown in Table 3, the majority of studies examining its relationship with wellbeing were conducted in the healthcare sector (50.0%), with nurses being the most frequently studied professionals (19.3%), followed by various other healthcare professionals (15.9%). The next most common context was multiple sectors (14.8%), followed by education, where teachers (8.0%) and university academic staff (1.1%) were examined. The military was the least studied sector (3.4%).

**TABLE 3:** Proportion of studies examining the relationship between psychological flexibility and wellbeing based on organisational sector and occupation.

Sector	Studies *n*	%	Occupation	Studies *n*	%
Healthcare	44	50.0%			
			Nurses	17	19.3%
			Various professions	14	15.9%
			Mental health practitioners	7	8.0%
			Physicians	6	6.8%
Multiple sectors	13	14.8%			
			Various professions	13	14.8%
Care & support work	7	8.0%			
			Community setting	5	5.7%
			Residential setting	2	2.3%
Education	8	9.1%			
			Teachers	7	8.0%
			University academic staff	1	1.1%
Service & technology	7	8.0%			
			Customer support staff	4	4.5%
			Various professions	2	2.3%
			Technology support staff	1	1.1%
Emergency & frontline services	6	6.8%			
			Police officers	3	3.4%
			Firefighters	1	1.1%
			Corrections officers	1	1.1%
			Emergency telecommunicators	1	1.1%
Military	3	3.4%	Military Personnel	3	3.4%

Finally, studies were conducted across a wide distribution of geographical regions (see Table 4). Studies from the United States were the most common (19.3%, *n* = 19), followed by the United Kingdom (14.8%, *n* = 13) and China (8.8%, *n* = 10).

**TABLE 4:** Studies published by geographical region.

Country	Number of studies	Country	Number of studies
Australia	4	Italy	3
Belgium	1	Japan	1
Brazil	1	Netherlands	4
Canada	1	Nigeria	1
China	10	Portugal	2
Colombia	2	South Korea	1
Finland	1	Spain	9
Germany	1	Sweden	4
Hong Kong^a^	1	Turkey	6
Switzerland^a^	1	United Kingdom	13
Ireland	1	United States	17

^a^ One study (Chong et al., 2023) occurred in two geographical regions.

## DISCUSSION

This scoping review provides a comprehensive synthesis of the literature on PF/PI and its relationship with wellbeing in an organisational context. In conducting this review, several broad themes emerged. First, studies examining PF/PI are increasing, and the rapid rise since 2020 indicates that it is an increasingly used, recognised and relevant construct in organisational settings. Second, the AAQ‐II is the most predominant measure, with increasing use of its context‐specific (i.e. workplace) derivatives. Third, the majority of studies are cross‐sectional. Fourth, existing research predominantly examines negative aspects of wellbeing, with burnout being the most frequently studied outcome. Finally, PF/PI is primarily examined in the healthcare sector and within Western countries. Each of these themes is discussed below.

### The rise of PF/PI

Our scoping review revealed that there is a growing number of studies examining the relationship between PF/PI and wellbeing in an organisational setting. Interestingly, 2024 (the year in which the scoping review was completed) had the highest number of published studies. This highlights the increased interest in the role of these constructs in organisations and aligns with the ongoing endeavour within organisational contexts to safeguard and support employee wellbeing (Hymel et al., 2011; Lu et al., 2022; Ogbonnaya et al., 2019). There is a growing body of evidence supporting the use of ACT for these purposes within organisations (Twohig et al., 2021), with PF/PI being the key mechanism of change within this therapy (Hayes et al., 2006). Encouragingly, the rapid rise in examining PF/PI reinforces the importance of conducting such a review to extrapolate key patterns and trends that are occurring in the literature.

### Measurement issues and PF/PI and dominance of AAQ‐II

Our review revealed considerable variation in how PF/PI is assessed within organisational studies. Most research continues to rely on the AAQ‐II (Bond et al., 2011). Although widely adopted, the AAQ‐II primarily reflects tendencies to struggle with thoughts or emotions rather than the broader behavioural processes emphasised in conceptualisations of PF (Chawla & Ostafin, 2007; Levin et al., 2014). A recurring issue is that the AAQ‐II is frequently interpreted as an indicator of PF, even though it was originally designed to assess patterns associated with PI (Hayes et al., 2006). Many organisational studies appear to treat flexibility and inflexibility as opposite ends of a single continuum; however, emerging work indicates that these processes do not always function as simple inverses (Doorley et al., 2020; Stabbe et al., 2019). Furthermore, concerns have also been raised about what the AAQ‐II captures (Doorley et al., 2020). Several investigations have demonstrated that AAQ‐II scores correlate strongly with negative affectivity, neuroticism and emotional distress, often more so than with theoretically related processes such as acceptance (Rochefort et al., 2018; Tyndall et al., 2019; Wolgast, 2014). If this pattern reflects substantive overlap, then AAQ‐II–based findings in workplace research may partly reflect general emotional vulnerability rather than the specific behavioural capacities central to PF theories (Doorley et al., 2020).

We also identified the use of context‐specific measures of the AAQ‐II. Most commonly, this was the Work‐Related Acceptance and Action Questionnaire. Rather than assessing broad patterns of responding, context‐specific variants are designed to capture how individuals react within a particular setting or demand, such as work‐related challenges (Ong et al., 2019). In a recent review, Ong et al. (2019) recommended that when using the AAQ‐II, it is beneficial to have both context‐specific variants and a general measure. It is pleasing to observe that, to some extent, this is occurring in the organisational literature. However, it must be noted, again, that some studies continue to conceptualise these context variants as measures of PF.

Finally, in regard to measures of PF/PI, we also observed a recent trend of increased use of other measures of PF/PI. This includes the use of multidimensional measures such as the MPFI, CompACT and PsyFlex. The creation of these measures has been motivated, in part, to overcome the issues that have arisen from the use of the AAQ‐II (see Cherry et al., 2021). It is promising to see an increased use of these measures when assessing PF/PI in organisations. However, the challenges in measuring the constructs of PF/PI must be noted. Kashdan et al. (2020) argued that more recent measures of PF (i.e. CompACT, MPFI and Psych Flex) lack conceptual alignment because they do not measure PF in the service of personally meaningful goals, which is central to PF definitions and theories (Hayes et al., 2011). Consequently, they developed the Personalized Psychological Flexibility Index (PPFI), which asks individuals to stipulate a specific valued goal they are currently working towards. PF is then assessed in relation to the pursuit of this goal. In a recent scoping review, Cherry et al. (2021) found that the PPFI was the superior measure of PF both in terms of psychometric quality and theoretical alignment. Our review found no studies using the PPFI to measure PF in relation to wellbeing within an organisational context. Furthermore, none of the multidimensional measures (MPFI, CompACT and PsyFlex) have been validated for workplace (i.e. context‐specific) measurement.

Given these ongoing measurement issues, we next examined how the AAQ‐II is defined and interpreted within the organisational literature. We found considerable inconsistency in how authors describe the construct assessed by the AAQ‐II. Some studies define it as a measure of experiential avoidance, whereas more recent work increasingly labels it as PI. However, a substantial number of studies continue to state that they are measuring PF despite using the AAQ‐II. As a result, the same instrument is variously described as assessing experiential avoidance, PI, PF, acceptance or even broader constructs such as psychological adaptability. This inconsistency is problematic because it obscures conceptual boundaries between PF, PI and related constructs. When the same measure is used to represent different theoretical processes, it becomes difficult to compare findings across studies or to determine whether observed effects reflect flexibility, inflexibility or broader distress‐related phenomena. Over time, this lack of clarity weakens theoretical precision and limits the interpretability and replicability of findings.

It is worth noting that although PF/PI in the clinical literature is developed via interventions, only 15.9% of wellbeing studies were intervention‐based studies. The majority of studies were of a cross‐sectional design (*n* = 68, 77.3%), used daily diary studies (*n* = 2, 2.3%) or adopted a longitudinal design (*n* = 3, 3.5%). This is important to note, as intervention research findings indicate the malleability of the PF/PI constructs, whereas cross‐sectional and daily diary studies suggest that PF/PI is being examined as a trait or state—a conceptual issue that has plagued other psychological wellbeing concepts such as mindfulness (Roche et al., 2021). Furthermore, the way in which researchers position PF/PI within analytic models reflects different theoretical assumptions about its function. For instance, beyond examining only direct bivariate relationships, 33 studies (37.5%) positioned PF/PI as either a mediating variable (*n* = 25) or a moderating variable (*n* = 11) within their models. Examining PF/PI as a stable trait (as implied by cross‐sectional correlational designs) conceptualises it as a relatively fixed individual difference characteristic. In contrast, positioning PF/PI as a mediator conceptualises it as a mechanism through which workplace conditions influence wellbeing, suggesting that it is malleable and, thus, a viable intervention target. This aligns more closely with ACT's conceptualisation of PF as a dynamic, trainable process through which individuals respond to stressors (Hayes et al., 2006). Thus, researchers are urged to ensure clarity as to whether they consider PF/PI a state, trait, intervention, practice or process in relation to understanding the wellbeing of the workforce.

### PF is often examined in relation to negative wellbeing

Finally, we also aimed to identify the wellbeing constructs examined in relation to PF/PI in an organisational context. This revealed that the literature focuses more on negative wellbeing (63.2%) than positive wellbeing (36.8%). Specifically, in regard to negative wellbeing, burnout was the most commonly examined construct. Burnout has been linked to a broad range of difficulties across psychological and physical domains, with research showing elevated vulnerability to mood‐related problems, sleep disruption, cardiovascular strain and diminished overall life satisfaction (Lizano, 2015; Nápoles, 2022; Rothenberger, 2017). Burnout is also associated with poorer functioning at work. Studies have documented links with difficulties sustaining performance, reduced efficiency, disruptions in judgement, diminished engagement with tasks and patterns of absence or turnover that place additional strain on organisations (Dewa et al., 2014; Michailidis & Banks, 2016; Mijakoski et al., 2015). Given its potential for profound implications within an organisational context, it is not surprising that this is a construct that the field will be eager to understand and modify through its relationship with PF/PI. Our scoping review also revealed that studies in the literature examine a broad range of negative wellbeing constructs, that is, depression, anxiety, sleep difficulties and alcohol use. This suggests that the literature is conducting a broad exploration of the relationship between PF and negative wellbeing, highlighting a growing interest in how this construct influences various dimensions of wellbeing.

Notably, emerging evidence continues to distinguish PF from inflexibility (Doorley et al., 2020; Malo et al., 2024). Consequently, this suggests that each of these constructs may have differing effects on wellbeing. For example, Howell and Demuynck (2021) examined the relationships between PF, inflexibility (measured using the MPFI) and wellbeing, both hedonic (pleasure and happiness) and eudaimonic (meaning and self‐realisation). The results indicated that both forms of wellbeing were independently associated with PF and inflexibility. Given this, increasing the focus on the relationship between PF and positive wellbeing would be beneficial, yet measurement needs to clearly assess PF, rather than inflexibility. This has the potential to provide unique and valuable insights into how individuals can thrive in various organisational settings. Understanding the role of PF in positive wellbeing can provide practical strategies for enhancing resilience, engagement and overall life satisfaction and supporting workers to thrive.

### PF, the healthcare sector and Westernised countries

This scoping review also provided an opportunity to extrapolate the specific organisational sectors in which the relationship between PF/PI and wellbeing is examined. In doing this, the vast majority of studies occurred in the healthcare sector. Nurses were the most commonly studied cohort, followed by a mixture of healthcare practitioners. Interestingly, the number of studies conducted on nurses (*n* = 17) surpassed most entire sectors. Research consistently shows that people working in healthcare face elevated risks to their psychological wellbeing compared with many other occupational groups (Barry et al., 2020; Lamiani et al., 2017; Palmer et al., 2022). Several interconnected demands make healthcare environments uniquely challenging for workers. These include the emotional intensity of caring for patients, sustained workloads, extended or irregular working hours, limited control over work conditions, organisational pressures and repeated exposure to distressing or traumatic events. Together, these factors place considerable strain on staff wellbeing (West et al., 2018). Given these challenges, it is encouraging that the literature is increasingly focused on understanding the factors influencing wellbeing in this context. However, it must be noted that several of these factors are not unique to the healthcare sector. We were surprised to find that relatively few studies have explored the relationship between PF/PI and wellbeing in sectors that face an elevated risk of wellbeing disruptions, for example, emergency and frontline services and the military (Kleim & Westphal, 2011; Lazar, 2014; Oster et al., 2017). The field would greatly benefit from exploring the relationship between PF/PI and wellbeing across a broader range of organisational settings.

Furthermore, our scoping review revealed that research in this field predominantly focuses on public organisational sectors, with participant recruitment reflecting this emphasis. Further, a lack of studies in competitive organisations—such as sales, marketing and logistics—was noted. These positions can often be associated with specific attributes and personality traits that support individuals to excel, and even thrive, in these roles. Future research in the field would therefore benefit from examining these constructs in a more nuanced, systemic way to determine if differences in their relationships exist.

Finally, the majority of studies come from traditional Western countries (e.g. the United States, the United Kingdom, Australia, Canada and Western Europe). China and Japan contributed a fair number of studies, illustrating some East Asian representation. However, there was a limited presence of studies from Latin America, Africa and the Middle East, suggesting underrepresentation of populations from these geographical regions. As national culture can shape mental wellbeing and workplace practices, a greater understanding of PF/PI in wider cultural contexts and geographical regions would aid the generalisability of the concept. For example, in collectivistic cultures, is valued living according to personal goals still valid for wellbeing at work? These issues are not exclusive to this field of study (Bou Zeineddine et al., 2022; Solomonov et al., 2024; Thalmayer et al., 2021), but they highlight the critical need for equity and representation in the constructs and relationships being examined.

## ALIGNMENT WITH ACT THEORY AND RECOMMENDATIONS FOR FUTURE RESEARCH

Encouragingly, the rapid rise in studies examining PF/PI reinforces the importance of conducting this scoping review to extrapolate key patterns and trends that are occurring in the literature. Although this review did not set out to evaluate the strength of associations between PF/PI and wellbeing, it is nonetheless possible to reflect on the extent to which organisational research aligns with ACT's theoretical model of PF. In many respects, the growing literature is consistent with the notion underlying ACT that PF/PI may impact wellbeing at work (Flaxman et al., 2023). However, several discrepancies suggest that workplace research has only partially translated ACT's theoretical foundations. First, the field's continued use of the AAQ‐II and its adaptations highlight the historical influence of this measure on organisational research. Although this approach has offered a practical starting point, it may not fully reflect the broader range of processes described in ACT models. The lack of uptake of theoretically consistent, multidimensional measures (e.g. MPFI, CompACT and PPFI) further contributes to this misalignment. Second, organisational studies frequently assume that flexibility and inflexibility represent a single linear dimension, yet both ACT theory and newer empirical work suggest they may function as related but distinct processes (Doorley et al., 2020; Stabbe et al., 2019). This conceptual simplification risks obscuring potentially important dynamics in workplace wellbeing. Third, the outcomes studied to date focus predominantly on distress and burnout, with relatively little attention given to positive indicators of wellbeing such as thriving, engagement or eudaimonic functioning. This contrasts with ACT's emphasis on values‐based living and flourishing.

Thus, although this review has offered valuable insights into the current state of PF/PI and workplace wellbeing research, it has also revealed important areas of theoretical misalignment. A further issue concerns whether PF/PI is best understood as a relatively stable, trait‐like characteristic, a state‐like process that fluctuates with daily demands or a malleable skill that can be cultivated through interventions. Organisational research has yet to examine these distinctions in detail, which limits our understanding of how PF/PI operates dynamically in workplace contexts. Building on these insights, we propose six recommendations to guide the field's continued development and advancement:

1. Future research should clearly distinguish PF, PI and experiential avoidance in both definition and measurement and avoid treating these constructs as interchangeable.
2. Prioritise longitudinal and experimental designs to clarify directionality and causal mechanisms linking PF/PI and wellbeing.
3. Researchers should explicitly specify whether PF/PI is conceptualised as a stable trait, a state‐like process or a modifiable intervention target, as these assumptions have important theoretical and methodological implications for understanding wellbeing at work.
4. Future studies should examine PF/PI across a broader range of occupational contexts, including lower‐stress and more competitive roles.
5. Research to date has predominantly focused on deficit‐based outcomes such as distress and burnout; future work should expand attention to positive wellbeing outcomes, including engagement, thriving and eudaimonic functioning, using measures that clearly assess PF rather than inflexibility.
6. Given the predominance of Western samples, future research should increase cultural and geographical diversity to better understand how PF/PI operates across different value systems and organisational contexts.

## LIMITATIONS AND CONCLUSIONS

The objective of this scoping review was to synthesise and understand the literature as it pertains to PF/PI and wellbeing in an organisational context. Therefore, this review did not examine *how* PF/PI is related to wellbeing, which is achieved through a systematic review or meta‐analysis where effect sizes and risks of bias are synthesised. Given the breadth of the studies our scoping review identified and the heterogeneity regarding methodologies, it would be challenging to systematically review these results this broadly. However, sufficient evidence exists to support this direction, and focusing on specific outcomes (i.e. resilience) or organisational sectors (i.e. education) is likely the most effective approach to achieving this. This aligns with a recent systematic review by Garner and Golijani‐Moghaddam (2021) on the relationship between work‐related quality of life and PF/PI in healthcare professionals. Additionally, because of the scoping review design of this study, we weighted studies similarly to present an overview of the research landscape. However, comparability across studies may be limited because of the lack of a study quality index. Further, although every effort was made to capture all published articles that met the inclusion criteria, there is always the possibility that relevant studies were missed. Although we selected the MEDLINE, PsycInfo, Scopus, Embase and Web of Science databases to provide broad coverage across the health, psychology and organisational literature, we acknowledge that additional databases (e.g. EBSCO Host, Emerald, ProQuest, PsycArticles, Sage, Taylor & Francis and Wiley) may also index relevant organisational psychology research. As such, although our approach maximised breadth and feasibility, it may not have captured every possible study in this domain.

Finally, being able to pursue important values in the face of obstacles and challenges is a worthy and important concept for organisational researchers regarding employee wellbeing. The workplace is an ecosystem within which a multitude of challenges continue to plague its participants. As such, PF/PI provides an interesting and much‐needed psychological resource for workers, and the recent increase in research suggests that the concept holds promise. Our scoping review identifies a number of areas of caution as the research grows, particularly as PF/PI is drawn from interventions (ACT). Thus, many areas of PF remain hazy within workplace wellbeing research.

In conclusion, this scoping review provides a synthesis and summary of studies that have examined the relationship between PF/PI and wellbeing in organisational settings. This review identified patterns and trends evident in the literature, and recommendations were made accordingly. It is evident that PF/PI is becoming an increasingly studied construct in organisations, and the history of ACT and PF/PI drawn from clinical work suggests it could be an important and much‐needed psychological resource from which employees can benefit. However, we suggest that workplace researchers proceed with caution. Ultimately, it is our hope that this scoping review will support the smooth progression and advancement of the area of PF/PI and wellbeing at work.

## CONFLICT OF INTEREST STATEMENT

The authors declare no conflicts of interest.

## ETHICS STATEMENT

Ethics is not required for systematic reviews.

## Supporting information


**Table S1.** Quick view frequent measures, items, dimensions for psychological flexibility and psychological inflexibility.
**Table S2.** A summary of the search terms used.
**Table S3.** Outcome measures: Positive general wellbeing.
**Table S4.** Outcome measures: Negative general wellbeing.
**Table S5.** Outcome measures: Positive occupational wellbeing.
**Table S6.** Studies examining psychological flexibility/inflexibility as mediators and/or moderators in organisational settings.

## Data Availability

The data that support the findings of this study are available from the corresponding author upon reasonable request.
