# Supplementary material for: A scoping review and research agenda: Psychological flexibility and wellbeing in organisations
Source: Appl Psychol Health Well Being. 2026 Mar 12;18(2):e70139. doi: 10.1111/aphw.70139 (PMC12981213; doi:10.1111/aphw.70139)
Supplement: Supplementary file 1 — Table S1. Quick view frequent measures, items, dimensions for psychological flexibility and psychological inflexibility. Table S2. A summary of the search terms used. Table S3. Outcome measures: Positive general wellbeing. Table S4. Outcome measures: Negative general wellbeing. Table S5. Outcome measures: Positive occupational wellbeing. Table S6. Studies examining psychological flexibility/inflexibility as mediators and/or moderators in organisational settings. [file APHW-18-0-s001.docx]

Supplementary Materials

Table S1. Quick View Frequent Measures, Items, Dimensions for Psychological Flexibility and Psychological Inflexibility

| **Measurements of Psychological Flexibility in Workplace Wellbeing Literature** | | | |
| --- | --- | --- | --- |
| **Year and Author** | **Measure Name** | **Measurement Definition** | **Measurement Dimensions** |
| 2004  Hayes et al. | Acceptance and Action Questionnaire (AAQ) | A measure of experiential avoidance and psychological inflexibility. | 9 items  Unidimensional measure |
| 2011  Frank et al. | AAQ-II | A variant of the AAQ above. Statements stem from either the likely dominance or nondominance of internal events in determining values-directed actions. | 7 items  Unidimensional measure |
| 2011  Bond et al. | Work-related Acceptance and Action Questionnaire (WAAQ) | A version of the AAQ-II in which the items represent psychological flexibility within the workplace. | 7 items  Unidimensional measure |
| 2015  Hinds et al. | Teacher Acceptance and Action Questionnaire (TAAQ) | A variant of the AAQ-II that includes a set of items reflecting experiential avoidance in teaching-specific contexts. | 10 items  Unidimensional measure |
| 2016  Fava & Bech | The Euthymia Scale—Psychological Flexibility (ES-PF) | Assesses psychological (in)flexibility and whether the person displays balance and resilience during changing or stressful situations. | 5 items  Unidimensional measure |
| 2016  Francis et al. | Comprehensive assessment of Acceptance and Commitment Therapy processes (CompACT) | Assesses psychological flexibility in terms of the three dyadic acceptance and commitment therapy (ACT) processes: (1) valued action, (2) openness to experience and (3) behavioural awareness. | 23 items  Unidimensional measure |
| 2021  Andrews et al. | Psy-Flex | The Psy-Flex draws from core skills found in the ACT measure of psychological flexibility | 6 items  Unidimensional measure |
| 2014  Ben-Itzhak et al. | Psychological Flexibility Questionnaire (PFQ) | Conceptualises psychological flexibility as a multifaceted construct that relates to the self and reality (environment). | Multidimensional  20 items in 5 dimensions:  1. Positive perception of change (5 items)  2. Characterisation of the self as flexible (5 items)  3. Self-characterisation as open and innovative (3 items)  4. Perception of reality as dynamic and changing (4 items)  5. Perception of reality as multifaceted (3 items) |
| 2014  Gamez et al. | Brief Experiential Avoidance Questionnaire (BEAQ) | A brief measure of experiential avoidance. Assesses the avoidance of pain, uneasiness, effort, upset, unpleasantness, discomfort, emotions, painful emotions, feelings, bad feelings, upsetting feelings, fear/anxiety, unpleasant memories and doubts. | Multidimensional  15 items in 4 dimensions:  1. Explicit avoidance behaviour (taken from the MEAQ Behavioural Avoidance, Distraction/Suppression and Procrastination subscales; 8 items)  2. Attitudes/beliefs regarding distress (taken from the MEAQ Distress Aversion subscale; 4 items)  3. Implicit avoidance (taken from the MEAQ Repression/ Denial subscale; 2 items)  4. Ability to respond effectively to distress (taken from the MEAQ Distress Endurance subscale; 1 item). |
| 2018  Rolffs et al. | Multi-dimensional Psychological Flexibility Inventory (MPFI) | The MPFI draws on the Hexaflex conceptual framework of ACT. The MPFI assesses the six dimensions of psychological inflexibility *and* the six dimensions of psychological flexibility based on ACT. | Multidimensional  60 items in 6 dimensions (PF/PI):  1. Acceptance/experiential avoidance (5 items)  2. Contact with the present moment/lack of contact with the present moment (5 items)  3. Self as context/content (5 items)  4. Defusion/ fusion (5 items)  5. Committed action/inaction (5 items)  6. Values/lack of contact with values (5 items) |
| 2020  Kashdan et al. | Personalised Psychological Flexibility Index (PPFI) | Developed to measure how people respond to uncomfortable internal states and external obstacles while pursuing valued goals (i.e., valued goals in relation to avoidance, acceptance and harnessing). | Multidimensional  15 items in 3 subscales:  1. Avoidance (5 items)  2. Acceptance (5 items)  3. Harnessing (5 items) |

Table S2. A summary of the search terms used.

|  | **Concept 1** | **Concept 2** | **Concept 3** |
| --- | --- | --- | --- |
| **Key concepts** | **Psychological Flexibility** | **Workplace Wellbeing** | **Population/Context** |
| **Free text terms / natural language terms** | Psychological flexibility; psychological inflexibility; cognitive fusion; Experiential Avoidance  *Tentative terms:*  ACT interventions/RTCs | Burnout; stress; work stress; stress management; psychological stress; resilience; emotion regulation; job satisfaction; coping; occupational stress; quality of life; compassion fatigue; Work Engagement; Wellbeing; Mental health; Depression  *Tentative terms:*  ? job performance, work ability, meeting goals | Organisational; organizational; employee; management; leadership; leader; workplace; work; staff; personnel |
| **Search terms** | “psychological flexibility” OR “psychological inflexibility” OR “ cognitive fusion” OR “experiential avoid*” | burnout OR “burn out” OR burn-out OR stress OR “work stress” OR “occupation* stress” OR “stress management” OR “psychological stress” OR resilien* OR “emotion regulat*” OR “job satisfaction” OR coping OR “occupational stress” OR “quality of life” OR “quality of working life” OR “compassion fatigue” OR “work engag*” OR wellbeing OR “Well being” OR well-being OR “psychological well being” OR “mental health” OR depress* | organisation* OR organization* OR employee* OR manage* OR leader* OR workplace OR work OR staff OR personnel OR workforce |
| **Controlled vocabulary** **terms / Subject terms**  (MeSH terms, Emtree terms)  *Consider: explode, major headings, subheadings* | *PSYC Info index terms:* “experiential avoidance” OR “cognitive flexibility” OR “Acceptance and Commitment Therapy”  *Medline MeSH terms*: “Acceptance and Commitment Therapy” | *PSYC Info index terms:* “well being” OR “quality of life” OR “quality of life measures” OR “compassion fatigue” OR “occupational stress” OR “quality of work life” OR stress OR “stress management”  *Medline MeSH terms*: “Burnout, Psychological” OR “Burnout, Professional” OR “Occupational Stress” OR “Quality of Life” OR “Emotional Regulation” OR “Job Satisfaction” OR “Depression” OR “Work Engagement” OR “Adaptation, Psychological” | *PSYC Info index terms:* “personnel management” OR “employees” OR “occupational groups” OR “human resources” OR “industrial-organizational psychology”  *Medline MeSH terms:* “Leadership” OR “Workplace” OR “Personnel Management” OR “Working Conditions” OR “Workforce” |

Table S3. Outcome measures: Positive general wellbeing.

| **Variable** | **Outcome Measure** | **Outcome Measure Citation** | **Authors** | **Studies Using Measure (n = )** |
| --- | --- | --- | --- | --- |
| **Coping** |  |  |  |  |
|  | Coping Self-Efficacy (CSE) | [Chesney et al., 2006](https://doi.org/10.1348/135910705X53155) | Omreore & Nwanzu, 2022 | 1 |
|  | Simplified Coping Style Questionnaire (SCSQ) | [Xie, 1998](https://psycnet.apa.org/record/1999-10962-018) | Yao et al., 2022 | 1 |
|  | Shortened Ways of Coping-Revised (SWC-R) | [Hatton & Emerson, 1995](https://psycnet.apa.org/doi/10.1111/j.1468-3148.1995.tb00160.x) | Kurz et al., 2014 | 1 |
|  | COPE Inventory | [Carver et al., 1998](https://doi.org/10.1037/t10027-000) | Arena et al., 2024 | 1 |
| **Curiosity** |  |  |  |  |
|  | Curiosity and Exploration Inventory-II (CEI-II) | [Kashdan et al., 2009](https://doi.org/10.1016/j.jrp.2009.04.011) | Arena et al., 2024 | 1 |
| **Emotion Regulation** |  |  |  |  |
|  | Difficulties with Emotion Regulation Scale (DERS) | [Gratz & Roemer, 2004](https://doi.org/10.1037/t01029-000) | Lilly and Allen, 2015 | 1 |
|  | Difficulties with Emotion Regulation Scale—Short Form (DERS-SF) | [Kaufman et al., 2016](https://doi.org/10.1007/s10862-015-9529-3) | Miller et al., 2022 | 1 |
|  | Short-Form Health Survey (SF-36; Emotion Functioning subscale) | [Ware & Sherbourne, 1992](https://doi.org/10.1097/00005650-199206000-00002) | McCracken and Yang, 2008 | 1 |
| **Happiness** |  |  |  |  |
|  | Subjective Happiness Scale (SHS) | [Lyubomirsky & Lepper, 1999](https://doi.org/10.1023/A:1006824100041) | Koydemir et al., 2023 | 1 |
|  | Work Affect Scale (WOKAS) | [Ferreira et al., 2008 [Google Scholar]](https://scholar.google.com/scholar_lookup?title=Desenvolvimento+e+valida%C3%A7%C3%A3o+de+uma+Escala+de+Afetos+no+Trabalho+(ESAFE)+%5BDevelopment+and+validation+of+a+Positive+and+Negative+Affect+Scale+(WOKAS)%5D&author=Ferreira+M.+C.&author=Silva+A.+P.&author=Fernandes+H.+A.&author=Almeida+S.+P&publication+year=2008&journal=Avalia%C3%A7%C3%A3o+Psicol%C3%B3gica&volume=7&pages=143-150) | Novaes et al., 2018 | 1 |
| **Life Satisfaction** |  |  |  |  |
|  | Life Satisfaction Questionnaire (LSQ) | [Carlsson & Hamrin, 2002](https://doi.org/10.1023/A:1015670628990) | Puolakanaho et al., 2018 | 1 |
|  | Satisfaction with Life Scale (SWLS) | [Diener, 1985](https://doi.org/10.1037/t01069-000) | Navarro‐Prados et al., 2024a; Proctor et al., 2024; Schéle et al., 2021; Tynan et al., 2022 | 4 |
| **Mindfulness** |  |  |  |  |
|  | Five Facet Mindfulness Questionnaire (FFMQ) | [Baer et al., 2006](https://doi.org/10.1037/t05514-000) | Galhardo et al., 2021; Puolakanaho et al., 2018; Waters et al., 2018 | 3 |
|  | Mindfulness Attention Awareness Scale (MAAS) | [Brown & Ryan, 2003](https://doi.org/10.1037/t04259-000) | Kent et al., 2019; Ramci et al., 2019; Wang et al., 2022b | 3 |
| **Perfectionism** |  |  |  |  |
|  | Clinical Perfectionism Questionnaire (CPQ) | [Fairburn et al., 2003](https://doi.org/10.1037/t59141-000) | van de Leur et al., 2024 | 1 |
| **Resilience** |  |  |  |  |
|  | Brief Resilience Scale (BRS) | [Smith et al., 2008](https://doi.org/10.1037/t51423-000) | Archer et al., 2024; Greville-Harris et al., 2024 | 2 |
|  | Brief Resilient Coping Scale (BRCS) | [Sinclair & Wallston, 2004](https://doi.org/10.1037/t08490-000) | Navarro-Prados et al., 2022; Navarro‐Prados et al., 2024a | 2 |
|  | Connor–Davidson Resilience Scale 10-Item (CD-RISC-10) | [Campbell-Sills & Stein, 2007](https://doi.org/10.1002/jts.20271) | Jiménez-Fernández et al., 2022; Johns et al., 2022; Kern et al., 2020; Young et al., 2021 | 4 |
|  | Connor–Davidson Resilience Scale 25-Item (CD-RISC-25) | [Connor & Davidson, 2003](https://doi.org/10.1002/da.10113) | Marchand et al., 2023 | 1 |
|  | Dispositional Resilience Scale (DRS-15) | [Bartone, 1995 [Google Scholar]](https://scholar.google.com/scholar_lookup?title=A%20Short%20Hardiness%20Scale&publication_year=1995&author=P.T.%20Bartone) | Blekić et al., 2023 | 1 |
|  | Resilience Scale (RS) | [Wagnild & Young, 1993 [APA PsycNet]](https://psycnet.apa.org/record/1996-05738-006) | Hendriks et al., 2021 | 1 |
| **Self-Compassion** |  |  |  |  |
|  | Self-Compassion Scale – Short Form (SCS-SF) | [Raes et al., 2011](https://doi.org/10.1037/t10179-000) | Clarke et al., 2014 | 1 |
| **Self-Efficacy (General)** |  |  |  |  |
|  | General Self-Efficacy Scale (GSES) | [Schwarzer & Jerusalem, 1995](https://doi.org/10.1037/t00393-000) | Brassey et al., 2020; Xu et al., 2017 | 2 |
| **Valuing** |  |  |  |  |
|  | Engaged Living Scale (ELS) | [Trompetter et al., 2013](https://doi.org/10.1037/t27748-000) | Kent et al., 2019 | 1 |
|  | Valuing Questionnaire (VQ) | [Snout at al., 2014](https://doi.org/10.1016/j.jcbs.2014.06.001) | Ulusoy and Çelik, 2022 | 1 |
|  | Work Values Questionnaire (WVQ) | [Avallone et al., 2010](https://psycnet.apa.org/record/2011-16354-006) | Vilardaga et al., 2011 | 1 |
| **Vitality** |  |  |  |  |
|  | Short-Form Health Survey (SF-36; Vitality subscale) | [Ware & Sherbourne, 1992](https://doi.org/10.1097/00005650-199206000-00002) | McCracken and Yang, 2008 | 1 |
|  | Subjective Vitality Scale (SVS) | [Ryan & Frederick, 1997](https://selfdeterminationtheory.org/wp-content/uploads/2014/04/1997_RyanFrederick.pdf) | Blanco-Donoso et al., 2019 | 1 |
| **Wellbeing** |  |  |  |  |
|  | General Well-Being Schedule (GWBS) | [Dupuy et al., 1978](https://doi.org/10.1037/t04083-000) | Wang et al., 2021 | 1 |
|  | Index of Wellbeing (IWB) | * Unable to retrieve citation | Tian et al., 2023 | 1 |
|  | Mental Health Continuum–Short Form (MHC–SF) | [Keyes, 2009](https://doi.org/10.1037/t30592-000) | Hendriks et al., 2021; Young et al., 2021 | 1 |
|  | Nurses Occupational Well-being Scale (NOWS) | [Chen et al., 2016 [Google Scholar]](https://scholar.google.com/scholar_lookup?journal=Chinese%20Journal%20of%20Modern%20Nursing&title=Initial%20establishment%20and%20reliability%20and%20validity%20testing%20for%20nurses%20occupational%20well%E2%80%90being%20questionnaire&author=L.%20Chen&author=H.%20Liu&author=Y.%20Y.%20Shang&author=X.%20Y.%20Shui&author=Q.%20Zhang&volume=22&issue=15&publication_year=2016&pages=2091-2096&) | Wang et al., 2022b | 1 |
|  | Psychological Wellbeing scale (PWS) | [Ryff & Keyes, 1995](https://doi.org/10.1037/0022-3514.69.4.719) | Omreore and Nwanzu, 2022 | 1 |
|  | Quality of Working Life Questionnaire for Cancer Survivors (QWLQ‑CS) | [de Jong et al., 2016](https://doi.org/10.1007/s11764-015-0485-4) | Proctor et al., 2024 | 1 |
|  | Scale of Psychological Well-Being | [Ryff et al., 1989](https://doi.org/10.1037/0022-3514.57.6.1069) | Puolakanaho et al., 2018 | 1 |
|  | *Authors' own scale - see article* | [Schéle et al., 2021](https://www.frontiersin.org/journals/psychology/articles/10.3389/fpsyg.2020.569605/full) | Schéle et al., 2021 | 1 |
|  | Short-Form Health Survey (SF-36; Psychological & Physical Quality of Life subscales) | [Ware & Sherbourne, 1992](https://doi.org/10.1097/00005650-199206000-00002) | McCracken and Yang, 2008 | 1 |
|  | World Health Organization Quality of Life (WHOQOL-BREF) | [Skevington et al., 2004](https://psycnet.apa.org/doi/10.1023/B:QURE.0000018486.91360.00) | Singh and O'Brien, 2020 | 1 |
|  | World Health Organization–Five Well-Being Index (WHO-5) | [World Health Organization, 2010](http://www.who-5.org/) | Baker et al., 2022; Merlo et al., 2024 | 1 |

Table S4. Outcome measures: Negative general wellbeing.

| **Variable** | **Outcome Measure** | **Outcome Measure Citation** | **Authors** | **Studies Using Measure (n = )** |
| --- | --- | --- | --- | --- |
| **Alcohol Use** |  |  |  |  |
|  | Alcohol Use Disorders Identification Test (AUDIT) | [Saunder et al., 1993](https://doi.org/10.1111/j.1360-0443.1993.tb02093.x) | Baker et al., 2022 | 1 |
| **Anger** |  |  |  |  |
|  | State-Trait Anger Expression Inventory-2 (STAXI-2) | [Spielberger, 1999](https://www.parinc.com/products/STAXI-2) | Lilly and Allen, 2015 | 1 |
|  |  |  |  |  |
| **Anxiety** |  |  |  |  |
|  | Beck Anxiety Inventory (BAI) | [Beck et al., 1988](https://doi.org/10.1037/t02025-000) | Aciksari and Karatepe, 2020 | 1 |
|  | COVID-19 Anxiety Scale (CAS) | [Silva et al., 2022](https://doi.org/10.1007/s12144-020-01195-0) | Jokić-Begić et al., 2020 | 1 |
|  | Death Attitude Profile-Revised (DAP-R; Death Anxiety subscale) | [Wong et al. 1994](https://doi.org/10.1037/t17237-000) | Arena et al., 2024 | 1 |
|  | Depression Anxiety Stress Scale – 21 (DASS-21) | [Lovibond & Lovibond, 1995](https://doi.org/10.1037/t01004-000) | Demïr et al., 2021; Galhardo et al., 2021; Greville-Harris et al., 2024; Paris et al., 2021 | 4 |
|  | Generalized Anxiety Disorder Scale–2 item (GAD-2) | [Spitzer et al., 2006](https://doi.org/10.1037/t02591-000) | Baker et al., 2022; Johns et al., 2022; Proctor et al., 2024 | 3 |
|  | Patient Health Questionnaire for Depression and Anxiety (PHQ-4) | [Kroenke et al., 2009](https://doi.org/10.1037/t06168-000) | Rodriguez-Rey 2024; Tynan et al., 2022 | 2 |
| **Burnout** |  |  |  |  |
|  | Bergen Burnout Inventory (BBI-15) | [Salmela-Aro et al., 2011](https://doi.org/10.1007/s00420-010-0594-3) | Puolakanaho et al., 2018 | 1 |
|  | Burnout Measure – Short Form (BMS) | [Malach-Pines, 2005](https://doi.org/10.1037/t00766-000) | Mojallal et al., 2021 | 1 |
|  | Copenhagen Burnout Inventory (CBI) | [Kristensen et al., 2005](https://doi.org/10.1080/02678370500297720) | El-Ashry et al., 2024; Greville-Harris et al., 2024; Malouf et al., 2023; Singh and O'Brien, 2020 | 4 |
|  | Job-Related Emotional Exhaustion Scale (JEES) | [Günay, 2021](https://doi.org/10.35379/cusosbil.828921) | Körük et al., 2023 | 1 |
|  | Karolinska Exhaustion Disorder Scale (KEDS) | [Besèr et al., 2014](https://doi.org/10.1111/sjop.12088) | van de Leur et al., 2024 | 1 |
|  | Maslach Burnout Inventory – Educators Survey (MBI-ES) | [Maslach et al., 1996](http://www.mindgarden.com/316-mbi-educators-survey) | Hinds et al., 2015; Koh et al., 2024 | 2 |
|  | Maslach Burnout Inventory – General Survey (MBI-GS) | [Maslach et al., 1996](https://www.mindgarden.com/312-mbi-general-survey) | Bravo et al., 2023; Bravo et al., 2021 | 2 |
|  | Maslach Burnout Inventory – Human Services Survey for Medical Personnel (MBI-HSS-MP) | [Maslach et al., 1996](https://www.mindgarden.com/315-mbi-human-services-survey-medical-personnel) | Zhao et al., 2023 | 1 |
|  | Maslach Burnout Inventory – Human Services Survey (MBI-HSS; Emotional exhaustion subscale) | [Maslach et al., 1996](https://www.mindgarden.com/314-mbi-human-services-survey) | Clarke et al., 2014 | 1 |
|  | Maslach Burnout Inventory (MBI) | [Maslach & Jackson, 1981](https://doi.org/10.1002/job.4030020205) | Archer et al., 2024; Barret and Stewart, 2020; Biglan et al., 2013; Chapman et al., 2024; Demïr et al., 2021; Inglesias et al., 2010; Jiménez-Fernández et al., 2022; Johns et al., 2022; McCracken and Yang, 2008*; Navarro-Prados et al., 2022; Navarro‐Prados et al., 2024a; Navarro‐Prados et al., 2024b; Sarabia-Cobo et al., 2021; Ulusoy and Çelik, 2022; Vilardaga et al., 2011; Yavuz et al., 2020 | 16 |
|  | Maslach Burnout Inventory Human Services Survey (MBI-HSS) | [Maslach et al., 1996](https://www.mindgarden.com/314-mbi-human-services-survey) | Noone and Hastings, 2011; Ortiz-Fune et al., 2020; Zarling et al., 2024 | 3 |
|  | Oldenburg Burnout Inventory (OLBI) | [Demerouti, 1999 [Google Scholar]](https://scholar.google.com/scholar?q=Demerouti,+E.Burnout+:+eine+folge+konkreter+arbeitsbedingungen+bei+dienstleistungs-+und+produktionst%C3%A4tigkeiten+1999.) | Paris et al., 2021; Slowiak and Jay, 2023 | 2 |
|  | Professional Quality of Life Scale Version 5 (ProQOL-5; Burnout subscale) | [Stamm (2010)](https://proqol.org/) | Duarte and Pinto-Gouveia, 2017; Gray and Rydon-Grange, 2020; Holding et al., 2024; Kent et al., 2019; Merlo et al., 2024 | 5 |
|  | Questionnaire of Burnout Syndrome in Nurses (NBS; Emotional Exhaustion subscale) | [Garrosa et al., 2008](https://doi.org/10.1016/j.ijnurstu.2006.09.003) | Blanco-Donoso et al., 2019 | 1 |
|  | Utrecht Burnout Scale – General Version (UBOS-A) | [Schaufeli & van Dierendonck, 2000 [Google Scholar]](https://scholar.google.com/scholar_lookup?hl=en&publication_year=2000&author=W.+B.+Schaufeli&author=D.+van+Dierendonck&title=%0A++++++++++++++++++%0A+++++++++++++++++++++UBOS%3A+Utrechtse+Burnout+Schaal%E2%80%94Handleiding+%5BUBOS%3A+Utrecht+Burnout+Scale%E2%80%94Manual%5D%0A+++++++++++++++) | Biron and van Veldhoven, 2012; Onwezen et al., 2014 | 2 |
| **Cognitive fusion** |  |  |  |  |
|  | Automatic Thoughts Questionnaire – Believability (ATQ-B) | [Netemeyer et al., 2002](https://doi.org/10.1177/0013164402062001008) | Puolakanaho et al., 2018 | 1 |
|  | Cognitive Fusion Questionnaire (CFQ) | [Gillandrs et al., 2014](https://doi.org/10.1016/j.beth.2013.09.001) | Kent et al., 2019; Miller et al., 2022; Wang et al., 2022a; Zhao et al., 2023 | 4 |
|  | Stigmatizing Attitudes—Believability Scale (SAB) | [Hayes et al., 2004](https://doi.org/10.1016/S0005-7894(04)80022-4) | Vilardaga et al., 2011 | 1 |
|  | Believability of Anxious Feelings and Thoughts Questionnaire (BAFT) | [Herzberg et al., 2012](https://doi.org/10.1037/a0027782) | Navarro-Prados et al., 2024 | 1 |
|  |  |  |  |  |
| **Depression** |  |  |  |  |
|  | Automatic Thoughts Questionnaire - Frequency (ATQ-F) | [Zettle & Hayes,1986](https://doi.org/10.1007/BF03392813) | Puolakanaho et al., 2018; Waters et al., 2018 | 2 |
|  | Beck Depression Inventory-II (BDI-II) | [Beck et al., 1996](https://doi.org/10.1037/t00742-000) | Aciksari and Karatepe, 2020; Gerhart et al., 2016; Lilly & Allen, 2015 | 3 |
|  | Center for Epidemiological Studies–Depression Scale (CES-D) | [Radloff, 1977](https://doi.org/10.1177/014662167700100306) | Biglan et al., 2013; Navarro-Prados et al., 2022; Navarro‐Prados et al., 2024a Yao et al., 2022; Yao et al., 2023 | 5 |
|  | Depression Anxiety Stress Scale – 21 (DASS-21) | [Lovibond & Lovibond, 1995](https://doi.org/10.1037/t01004-000) | Demïr et al., 2021; Galhardo et al., 2021; Greville-Harris et al., 2024; Paris et al., 2021; Wang et al., 2022a | 5 |
|  | Patient Health Questionnaire – 2 (PHQ-2) | [Kroenke et al., 1995](https://doi.org/10.1037/t01004-000) | Baker et al., 2022 | 1 |
|  | Patient Health Questionnaire – 8 (PHQ-8) | [Kroenke et al., 1999](https://doi.org/10.1037/t06165-000) | Hinds et al., 2015; Johns et al., 2022 | 2 |
|  | Patient Health Questionnaire – 9 (PHQ-9) | [Kroenke et al., 1999](https://doi.org/10.1037/t06165-000) | Ahn et al., 2024; Bryan et al., 2015; Proctor et al., 2024; Tian et al., 2023 | 4 |
|  | Patient Health Questionnaire for Depression and Anxiety (PHQ-4) | [Kroenke et al., 2009](https://doi.org/10.1037/t06168-000) | Rodríguez-Rey et al., 2024; Tynan et al., 2022 | 2 |
|  | Positive and Negative Affect Schedule – Short Form (PANAS-SF) | [Thompson, 2007](https://psycnet.apa.org/doi/10.1177/0022022106297301) | Mojallal et al., 2021 | 1 |
|  | Work Affect Scale (WOKAS) | [Ferreira et al., 2008 [Google Scholar]](https://scholar.google.com/scholar_lookup?title=Desenvolvimento+e+valida%C3%A7%C3%A3o+de+uma+Escala+de+Afetos+no+Trabalho+(ESAFE)+%5BDevelopment+and+validation+of+a+Positive+and+Negative+Affect+Scale+(WOKAS)%5D&author=Ferreira+M.+C.&author=Silva+A.+P.&author=Fernandes+H.+A.&author=Almeida+S.+P&publication+year=2008&journal=Avalia%C3%A7%C3%A3o+Psicol%C3%B3gica&volume=7&pages=143-150) | Novaes et al., 2018 | 1 |
| **Fatigue** |  |  |  |  |
|  | Fatigue Scale (FS-14) | [Zuoji, 2015](https://onlinelibrary.wiley.com/doi/book/10.1002/9781118453940%20%5b2022-07-13%5d) | Wang et al., 2022a | 1 |
|  | Fatigue Assessment Instrument (FAI) | [Schwartz, 1993](https://doi.org/10.1037/t22082-000) | Yao et al., 2023 | 1 |
| **Intolerance of Uncertainty** |  |  |  |  |
|  | Intolerance of Uncertainty Scale–12 (IUS-12) | [Carleton et al., 2007](https://doi.org/10.1016/j.janxdis.2006.03.014) | Johns et al., 2022; Malouf et al., 2023 | 2 |
|  | Physicians Reaction to Uncertainty scale (PRU) | [Gerrity et al., 1995](https://doi.org/10.1007/BF02250510) | Malouf et al., 2023 | 1 |
| **Obsessive Compulsive Disorder (OCD)** |  |  |  |  |
|  | Obsessive-Compulsive Inventory revised (OCI-R) | [Foa et al., 2002](https://psycnet.apa.org/doi/10.1037/1040-3590.14.4.485) | Wang et al., 2024 | 1 |
| **Psychological distress** |  |  |  |  |
|  | General Health Questionnaire-12 (GHQ-12) | [Goldberg, 1972](https://doi.org/10.1037/t00297-000) | Barret and Stewart, 2020; Bond and Flaxman, 2006; Bond et al., 2008; Bond et al., 2013; Bravo et al., 2023; Gray and Rydon-Grange, 2020; Holmberg et al., 2020; Waters et al., 2018; Xu et al., 2017; | 9 |
|  | Kessler Psychological Distress Scale (K10) | [Kessler et al., 2002](https://doi.org/10.1037/t08324-000) | Yildirim 2024 | 1 |
|  | Positive and Negative Affect Schedule (PANAS) | [Watson et al., 1988](https://doi.org/10.1037/0022-3514.54.6.1063) | Marchand et al., 2023 | 1 |
|  | Short-Form Health Survey (SF-36; Psychological distress subscale) | [Ware & Sherbourne, 1992](https://doi.org/10.1097/00005650-199206000-00002) | McCracken and Yang, 2008 | 1 |
| **Post Traumatic Stress Disorder (PTSD)** |  |  |  |  |
|  | Abbreviated PTSD Checklist (PCL-6) | [Lang et al., 2012](https://doi.org/10.%201016/j.genhosppsych.2012.02.003) | Baker et al., 2022 | 1 |
|  | Primary Care PTSD screen for DSM-5 (PC-PTSD-5) | [Prins et al., 2016](https://www.ptsd.va.gov/) | Rodríguez-Rey et al., 2024; Tynan et al., 2022 | 2 |
|  | Professional Quality of Life Scale Version 5 (ProQOL-5; Secondary traumatic stress subscale) | [Stamm, 2010](https://proqol.org/) | Merlo et al., 2024 | 1 |
|  | PTSD Checklist – Military Version (PCL-M) | Weathers et al., 2013 | Bryan et al., 2015 | 1 |
|  | PTSD Checklist for DSM-5 (PCL-5) | [Weathers et al., 2013](https://www.ptsd.va.gov/) | Blekić et al., 2023 | 1 |
|  | PTSD Checklist—Civilian Version (PCL-C) | Weathers et al., 2013 | Gerhart et al., 2016; Johns et al., 2022; Lilly and Allen, 2015; Miller et al., 2022; Tian et al., 2023 | 5 |
|  | Secondary Traumatic Stress Scale (STSS) | [Bride et al., 2004](https://doi.org/10.1037/t06768-000) | Chapman et al., 2024; Gray and Rydon-Grange, 2020 | 2 |
|  | Moral Injury Events Scale (MIES) | [Nash et al., 2013](https://www.ptsd.va.gov/) | Ulusoy and Çelik, 2022 | 1 |
| **Shame** |  |  |  |  |
|  | Personal Feelings Questionnaire-2 (PFQ-2) | [Harder & Lewis, 1987 [APA PsycNet]](https://psycnet.apa.org/record/1987-97182-006) | Singh and O'Brien, 2020 | 1 |
|  |  |  |  |  |
| **Sleep difficulties** |  |  |  |  |
|  | Dysfunctional Beliefs and Attitudes about Sleep-16 (DBAS-16) | [Morin et al., 2007](https://doi.org/10.1093/sleep/30.11.1547) | Ahn et al., 2024 | 1 |
|  | Glasgow Sleep Effort Scale (GSES) | [Broomfield & Espie, 2005](https://doi.org/10.1037/t67027-000) | Ahn et al., 2024 | 1 |
|  | Pittsburgh Sleep Quality Index (PSQI) | [Buysee et al., 1989](https://doi.org/10.1037/t05178-000) | Wang et al., 2024 | 1 |
|  | Insomnia Severity Index (ISI) | [Morin, 1993](https://doi.org/10.1037/t07115-000) | Ahn et al., 2024; van de Leur et al., 2024 | 2 |
| **Stress** |  |  |  |  |
|  | Depression Anxiety Stress Scale – 21 (DASS-21; stress subscale) | [Lovibond & Lovibond, 1995](https://doi.org/10.1037/t01004-000) | Demïr et al., 2021; Galhardo et al., 2021; Greville-Harris et al., 2024; Kurz et al., 2014; Paris et al., 2021; Ulusoy and Çelik, 2022; Young et al., 2021 | 7 |
|  | Perceived Stress Scale - 10 (PSS-10) | [Cohen et al., 1983](https://doi.org/10.1037/t02889-000) | Holmberg et al., 2019; Holmberg et al., 2020; Puolakanaho et al., 2018 | 3 |
|  | Perceived Stress Scale - 14 (PSS-14) | [Cohen et al., 1983](https://doi.org/10.1037/t02889-000) | Barret and Stewart, 2020; Blekić et al., 2023; Ding and Wang, 2022; Kent et al., 2019; Navarro-Prados et al., 2024; Zhao et al., 2023 | 6 |
|  | Perceived Stress Scale - 4 (PSS-4) | [Cohen et al., 1983](https://doi.org/10.1037/t02889-000) | Tynan et al., 2022 | 1 |
| **Suicidality** |  |  |  |  |
|  | Suicidal Behaviors Questionnaire-Revised (SBQ-R) | [Osman et al., 2001](https://doi.org/10.1037/t14542-000) | Bryan et al., 2015 | 1 |
| **Worry** |  |  |  |  |
|  | Penn State Worry Questionnaire (PSWQ) | [Meyer et al., 1990](https://doi.org/10.1037/t01760-000) | Koh et al., 2024 | 1 |
|  | Penn State Worry Questionnaire ultra-brief (PSWQ-brief) | [Berle et al., 2010](https://doi.org/10.1002/cpp.724) | van de Leur et al., 2024 | 1 |

**Note**: * McCracken and Yang (2008) used 3 items of the Maslach Burnout Inventory (MBI)

Table S5. Outcome measures: Positive occupational wellbeing.

| **Variable** | **Outcome Measure** | **Outcome Measure Citation** | **Authors** | **Studies Using Measure (n = )** |
| --- | --- | --- | --- | --- |
| **Autonomous Motivation** |  |  |  |  |
|  | Personal Projects Questionnaire (PPQ) | [Kasser, 1998](https://doi.org/10.1177/01461672982412006) | Arena et al., 2024 | 1 |
|  | Intrinsic Job Motivation | [Warr et al., 1979](https://doi.org/10.1111/j.2044-8325.1979.tb00448.x) | Bond et al., 2008; Bond et al., 2013 | 2 |
| **Compassion satisfaction** |  |  |  |  |
|  | Professional Quality of Life Scale Version 5 (Pro-QOL-5; Compassion Satisfaction subscale) | [Stamm, 2010](https://img1.wsimg.com/blobby/go/dfc1e1a0-a1db-4456-9391-18746725179b/downloads/ProQOL%20Manual.pdf?ver=1622839353725) | Duarte and Pinto-Gouveia, 2017; Gray and Rydon-Grange, 2020; Holding et al., 2024; Kent et al., 2019; Merlo et al., 2024; Sarabia-Cobo et al., 2021 | 6 |
|  |  |  |  |  |
| **Job Performance** |  |  |  |  |
|  | Individual Work Performance Questionnaire (IWPQ) | [Koopmans et al., 2014](https://doi.org/10.1037/t35489-000) | Archer et al., 2024 | 1 |
|  | Job performance scale (JPS) | [Çalışkan & Köroğlu, 2022](https://doi.org/10.29131/uiibd.1201880) | Ding and Wang, 2022; Yildirim, 2024 | 2 |
|  | Performance on customer service software (Learning) | [Bond and Flaxman, 2006](https://doi.org/10.1300/J075v26n01_05) | Bond and Flaxman, 2006 | 1 |
|  | Performance-to-target ratio (Performance) | [Bond and Flaxman, 2006](https://doi.org/10.1300/J075v26n01_05) | Bond and Flaxman, 2006 | 1 |
|  | Task-Based Job Performance scale (TBJPS) | [Goodman & Svyantek, 1999](https://doi.org/10.1006/jvbe.1998.1682) | Onwezen et al., 2014 | 1 |
|  | World Health Organization and Performance Questionnaire (WHO‐HPQ) | [Kessler et al. 2004](https://doi.org/10.1097/01.jom.0000126683.75201.c5) | Mojallal et al., 2021 | 1 |
| **Occupational Self efficacy** |  |  |  |  |
|  | Teacher Efficacy Scale (TES) | [Gibson & Dembo, 1984](https://doi.org/10.1037/t03431-000) | Biglan et al., 2013 | 1 |
|  | Occupational Self-Efficacy Scale (OSES) | [Schyns & Collani, 2002](https://doi.org/10.1080/13594320244000148) | Schéle et al., 2021 | 1 |
| **Sense of Coherence** |  |  |  |  |
|  | Sense of Coherence 13-item scale (SOC-13) | [Antonovsky, 1987 [APA PsycNet]](https://psycnet.apa.org/record/1987-97506-000) | Navarro-Prados et al., 2024 | 1 |
| **Work Engagement** |  |  |  |  |
|  | Utrecht Work Engagement Scale (UWES-17) | [Schaufeli et al., 2002](https://doi.org/10.1037/t07164-000) | Bond et al., 2013; Blanco-Donoso et al., 2019; Bravo et al., 2023; Galhardo et al., 2021; Holmberg et al., 2019; Holmberg et al., 2019; Holmberg et al., 2020; Wang et al., 2021; Xu et al., 2017 | 8 |
|  | Utrecht Work Engagement Scale-Short (UWES-S) | [Shimazu et al., 2008](https://doi.org/10.1111/j.1464-0597.2008.00333.x) | Koh et al., 2024 | 1 |

Table S5. Outcome measures: Negative occupational wellbeing.

| **Variable** | **Outcome Measure** | **Outcome Measure Citation** | **Authors** | **Studies Using Measure (n = )** |
| --- | --- | --- | --- | --- |
| **Compassion fatigue** |  |  |  |  |
|  | Compassion Fatigue Scale-Revised (CFS-R) | [Adams et al., 2006](https://doi.org/10.1037/0002-9432.76.1.103) | Eichorst et al., 2024 | 1 |
|  | Professional Quality of Life Scale Version 5 (Pro-QOL-5; Compassion fatigue subscale) | [Stamm, 2010](https://img1.wsimg.com/blobby/go/dfc1e1a0-a1db-4456-9391-18746725179b/downloads/ProQOL%20Manual.pdf?ver=1622839353725) | Duarte and Pinto-Gouveia, 2017; Holding et al., 2024; Kent et al., 2019; Sarabia-Cobo et al., 2021 | 4 |
| **Emotional dissonance** |  |  |  |  |
|  | Perth Emotional Labor Scale (PELS, Emotional Dissonance factor) | [Andela et al., 2015](https://doi.org/10.1037/cbs0000024) | Clarke et al., 2014 | 1 |
|  | Surface Acting and Deep Acting Scale (DASAS) | [Grandey, 2003](https://doi.org/10.5465/30040678) | Biron and van Veldhoven, 2012 | 1 |
|  | Emotional Labour Scale (ELS) | [Brotheridge & Lee, 2003](https://doi.org/10.1348/096317903769647229) | Biron and van Veldhoven, 2012 | 1 |
| **Imposterism** |  |  |  |  |
|  | Imposterism Scale | [Leary et al., 2001](https://doi.org/10.1111/1467-6494.00114) | Malouf et al., 2023 | 1 |
| **Intention to leave** |  |  |  |  |
|  | Nurses’ Intention to Leave the Nursing Profession Questionnaire (NILNPQ) | [Mohamed & Mohamed, 2013](https://www.lifesciencesite.com/lsj/life1002/035_17059life1002_223_229.pdf) | El-Ashry et al., 2024 | 1 |
| **Occupational stress** |  |  |  |  |
|  | Chinese Nursing Stress Scale (CNSS) | [Li & Liu, 2000 [Google Scholar]](https://scholar.google.com/scholar_lookup?&title=Job%20Stressors%20and%20Burnout%20among%20Staff%20Nurses&journal=Chin%20J%20Nurs&volume=35&issue=11&pages=645-649&publication_year=2000&author=Li%2CXM&author=Liu%2CYJ) | Wang et al., 2021 | 1 |
|  | Index of Teaching Stress (ITS) | [Greene et al., 1997](https://doi.org/10.1016/S0022-4405(97)00006-X) | Biglan et al., 2013 | 1 |
|  | Occupational Stress Indicator (OSI) | [Cooper et al., 1988](https://doi.org/10.1037/t12433-000) | Ramci et al., 2019 | 1 |
|  | Occupational Stress Inventory-Revised (OSI-R) | [Osipow, 1998](https://www.parinc.com/products/OSI-R) | Quan et al., 2022 | 1 |
|  | Perceived occupational stress (POS) | [Marcatto et al., 2022](https://doi.org/10.1027/1015-5759/a000677) | Yildirim et al., 2024 | 1 |
|  | Professional Life Stress Scale (PLSS) | [Fontana, 1989 [Google Scholar]](https://scholar.google.com/scholar_lookup?title=Professional%20life%20stress%20scale%20%20managing%20stress&publication_year=1989&author=D.%20Fontana) | Aciksari and Karatepe, 2020 | 1 |
|  | Staff Stressor Questionnaire (SSQ) | [Hatton et al., 1999](https://doi.org/10.1016/S0891-4222(99)00009-8) | Holding et al., 2024; Kurz et al., 2014 | 2 |
| **Work disability** |  |  |  |  |
|  | Work Ability Index (WAI) | [Tuomi et al., 1991](https://doi.org/10.1037/t58607-000) | Puolakanaho et al., 2018 | 1 |
| **Workplace ostracism** |  |  |  |  |
|  | Workplace Ostracism Scale (WOS) | [Ferris et al., 2008](https://doi.org/10.1037/a0012743) | Ding and Wang, 2022 | 1 |

Table S6. Studies examining psychological flexibility/inflexibility as mediators and/or moderators in organisational settings

| Study | Occupational Group | Analytic Role | PF / PI | Key Pathways Examined |
| --- | --- | --- | --- | --- |
| Blanco-Donoso et al. (2019) | Nurses | Mediation | PF | Social support → PF → Vigor, emotional exhaustion, vitality |
| Chong et al. (2022) | Nurses | Mediation (SEM) | PF | Job satisfaction → PF → Mental wellbeing; Burnout → PF → Mental health problems |
| Greville-Harris et al. (2024) | Physicians (surgeons) | Mediation | PF | Personality traits → PF / resilience → Mental health outcomes |
| Hendriks et al. (2021) | Private-sector employees | Mediation | PF | Intervention → PF → Mental wellbeing, resilience |
| Holding et al. (2024) | Disability support workers | Mediation | PF | Work stress → PF → Burnout, compassion fatigue, compassion satisfaction |
| Kern et al. (2020) | Teachers | SEM | PF | PF → Teaching resiliency → Sense of purpose → Intent to remain teaching |
| Körük et al. (2023) | White-collar workers | Mediation (SEM) | PF | Job satisfaction / emotional exhaustion → PF → Dyadic marital adjustment |
| van de Leur et al. (2024) | Various occupations | Mediation (LMM, RI-CLPM) | PF | Time in treatment → PF → Exhaustion; PF alongside sleep, worry, perfectionism |
| Wang et al. (2024) | Nurses | Mediation | PF | Obsessive–compulsive symptoms → PF → Sleep quality |
| Waters et al. (2018) | Healthcare workers | Mediation (bootstrapped) | PF | ACT → PF, mindfulness, defusion → Psychological distress |
| Yao et al. (2023) | Nurses | Mediation (bootstrapped) | PF | Fatigue-related variables → PF → Depression |
| Yildirim et al. (2024) | Teachers | Mediation | PF | Occupational stress → PF + meaning → Job satisfaction, performance, distress |
| Rodríguez-Rey et al. (2024) | Healthcare workers | Mediation (SEM) | PF / PI | Work stressors → PI, resilience → Depression, anxiety, PTSD |
| Singh & O’Brien (2020) | Various occupations | Mediation (SEM) | PF | Workplace stressors → PF → Physical & psychological wellbeing |
| Holmberg et al. (2020) | Healthcare workers | Mediation (SEM; longitudinal) | PF | Distress → PF → Work engagement; ΔPF → ΔEngagement |
| Proctor et al. (2024) | Cancer-survivor employees | Mediation | PF | Work quality → PF subprocesses → Wellbeing outcomes |
| Bond et al. (2008) | Call-centre employees | Mediated moderation | PF | Intervention × PF → Job control → Distress, absenteeism |
| Clarke et al. (2014) | Psychologists | Path analysis | PF | Emotional dissonance × PF → Emotional exhaustion |
| Kang et al. (2023) | Air-force pararescue | Moderation | PF | Burnout × PF → Insomnia |
| Novaes et al. (2018) | Various occupations | Moderation (SEM) | PF | Job demands/resources × PF → Job satisfaction & affect |
| Omreore & Nwanzu (2022) | Academic staff | Moderated mediation | PF | Work–leisure conflict → Wellbeing (moderated by PF) |
| Ramci et al. (2019) | Healthcare workers | Moderation (SEM) | PF | Managerial & intrinsic factors × PF → Health outcomes |
| Biron & van Veldhoven (2012) | Non-profit staff | Moderation (multilevel) | PF | Emotional demands × PF → Daily emotional exhaustion |
| Bryan et al. (2015) | Air-force personnel | Moderation | PF | Depression/PTSD × PF → Suicidal ideation |
| Onwezen et al. (2014) | Non-profit employees | Moderation & moderated mediation | PF | Emotional demands × PF → Exhaustion → Performance |
| Baker et al. (2022) | Police officers | Mediation & moderation (SEM) | PF | PTSS × PF → Psychiatric symptoms, alcohol use |
| Slowiak & Jay (2023) | ABA practitioners | Mediation & moderation | PF | Work demands/support → PF → Burnout |
| Hinds et al. (2015) | Teachers | Mediation | PI | Teacher stress → PI → Burnout, depression |
| Koh et al. (2024) | Teachers | Mediation | PI | Occupational stressors → PI → Exhaustion, depersonalisation |
| Kurz et al. (2014) | Disability support workers | Mediation | PI | Workplace stressors → PI → Psychological distress |
| Wang et al. (2022a) | Nurses | Serial mediation | PI | Mental fatigue → PI → Cognitive fusion → Negative emotions |
| Koydemir et al. (2023) | Managers/Subordinates | Mediation | PI | Leaders’ PI → Followers’ PI, need frustration |
| Eichorst et al. (2024) | Care workers | Moderation | PI | Death exposure × PI → Compassion fatigue |

Note. PF = psychological flexibility; PI = psychological inflexibility.
